# Cyclin E expression is associated with high levels of replication stress in triple-negative breast cancer

**DOI:** 10.1038/s41523-020-00181-w

**Published:** 2020-09-07

**Authors:** Sergi Guerrero Llobet, Bert van der Vegt, Evelien Jongeneel, Rico D. Bense, Mieke C. Zwager, Carolien P. Schröder, Marieke Everts, Rudolf S. N. Fehrmann, Geertruida H. de Bock, Marcel A. T. M. van Vugt

**Affiliations:** 1 Department of Medical Oncology, University of Groningen, University Medical Center Groningen, Groningen, The Netherlands; 2 Department of Pathology and Medical Biology, University of Groningen, University Medical Center Groningen, Groningen, The Netherlands; 3 Department of Epidemiology, University of Groningen, University Medical Center Groningen, Groningen, The Netherlands

**Keywords:** Breast cancer, Breast cancer

## Abstract

Replication stress entails the improper progression of DNA replication. In cancer cells, including breast cancer cells, an important cause of replication stress is oncogene activation. Importantly, tumors with high levels of replication stress may have different clinical behavior, and high levels of replication stress appear to be a vulnerability of cancer cells, which may be therapeutically targeted by novel molecularly targeted agents. Unfortunately, data on replication stress is largely based on experimental models. Further investigation of replication stress in clinical samples is required to optimally implement novel therapeutics. To uncover the relation between oncogene expression, replication stress, and clinical features of breast cancer subgroups, we immunohistochemically analyzed the expression of a panel of oncogenes (Cyclin E, c-Myc, and Cdc25A,) and markers of replication stress (phospho-Ser33-RPA32 and γ-H2AX) in breast tumor tissues prior to treatment (*n* = 384). Triple-negative breast cancers (TNBCs) exhibited the highest levels of phospho-Ser33-RPA32 (*P* < 0.001 for all tests) and γ-H2AX (*P* < 0.05 for all tests). Moreover, expression levels of Cyclin E (*P* < 0.001 for all tests) and c-Myc (*P* < 0.001 for all tests) were highest in TNBCs. Expression of Cyclin E positively correlated with phospho-RPA32 (Spearman correlation *r* = 0.37, *P* < 0.001) and γ-H2AX (Spearman correlation *r* = 0.63, *P* < 0.001). Combined, these data indicate that, among breast cancers, replication stress is predominantly observed in TNBCs, and is associated with expression levels of Cyclin E. These results indicate that Cyclin E overexpression may be used as a biomarker for patient selection in the clinical evaluation of drugs that target the DNA replication stress response.

## Introduction

Breast cancers are the most frequently diagnosed neoplasms worldwide, with approximately 1.38 million women being diagnosed with breast cancer worldwide every year. One-third of these women subsequently die of this disease, accounting for ~14% of all cancer-related deaths in women^1^. Therefore, there is an urgent clinical need for improved breast cancer treatment.

Breast cancers are very heterogeneous, and multiple classification methods have been developed to stratify patient groups. Using gene expression profiling, at least six major breast cancer subgroups have been defined, including “normal-like”, “luminal A”, “luminal B”, “HER2-enriched”, “claudin-low”, and “basal-like”^2^. Furthermore, combining copy number variations with gene expression analysis allowed identification of ten clusters that are associated with differential clinical outcome^3^. In standard care, breast cancers are subtyped based on the expression of the estrogen and progesterone receptors (ER and PR) and the human epidermal growth factor receptor-2 (HER2), as these receptors are “oncogenic drivers” and relevant drug targets. Patients with breast cancers that do not express the ER, PR, and HER2, so-called triple-negative breast cancers (TNBCs), do not benefit from antihormonal or anti-HER2-targeted treatments, and rely on conventional chemotherapeutic regimens. Initially, high response rates to conventional chemotherapeutics are seen in TNBC, however, tumors often recur and women have a poor prognosis overall^4^. TNBCs display aggressive behavior, and account for ~15–20% of all invasive breast cancers^4^.

TNBC tumors show a large degree of overlap with the intrinsic “basal‐like” and “claudin‐low” subgroups, lack common “druggable” aberrations, but share a profound genomic instability^5^. Finding novel treatment options for such genomically instable cancers is not only relevant for TNBCs, but also for other hard-to-treat cancers with extensive genomic instability, including ovarian and pancreatic cancers^6,7^.

Evidence is increasingly pointing to “replication stress” as a driver of genomic instability^8,9^. DNA replication is initiated at certain genomic loci called “replication origins”^9^. Replication origins are fired in a temporally controlled way, which prevents exhaustion of the nucleotide pool. A key source of replication stress in cancer cells appears to be the uncoordinated firing of replication origins due to oncogene activation^8–11^. As a consequence, oncogene activation depletes the nucleotide pool, leading to slowing or complete stalling of replication forks^12^. Oncogenes that have been linked to the induction of replication stress are the transcription factor c-Myc^13^ and Cyclin E, which acts in conjunction with cyclin-dependent kinase-2 (CDK2) to promote S-phase entry. It was shown that Cyclin E overexpression triggers aberrant origin firing with consequent nucleotide pool depletion, leading to replication fork stalling and genomic instability^12,14^. Likewise, overexpression of the Cdc25A phosphatase, which activates CDK2, promotes premature cell cycle progression and genomic instability^15–17^.

Cells are equipped with multiple mechanisms to survive replication stress. During replication fork stalling, single-stranded DNA (ssDNA) is exposed and rapidly activates the so-called “replication checkpoint”, in which the ATR kinase is the central player^18^. Activation of the replication checkpoint facilitates the rapid coating of ssDNA at replication forks with replication protein-A (RPA), which is phosphorylated by ATR^19,20^. When stalled replication forks are not resolved in time, they can collapse and cause DNA double-strand breaks (DSBs), triggering phosphorylation of the histone variant H2AX at serine 139, which is referred to as γ-H2AX^21^.

Genomically instable tumors increasingly rely for their survival on mechanisms that allow cells to resolve replication stress-induced DNA lesions, including cell cycle checkpoints^22^. Hence, cell cycle checkpoint kinases, including WEE1 and ATR, are potential therapeutic targets for tumors with high levels of replication stress. In order to implement novel therapeutic agents that target tumors with high levels of replication stress optimally, it is essential to know which tumor subgroups display replication stress. To this end, and to find potential biomarkers for tumors with high levels of replication stress, we examined replication stress levels in relation to oncogene expression and clinicopathological data in a consecutive well-defined series of breast cancer samples.

## Results

### Overexpression of Cyclin E1 results in replication stress and increased sensitivity to ATR and WEE1 inhibition

**Fig. 1:**
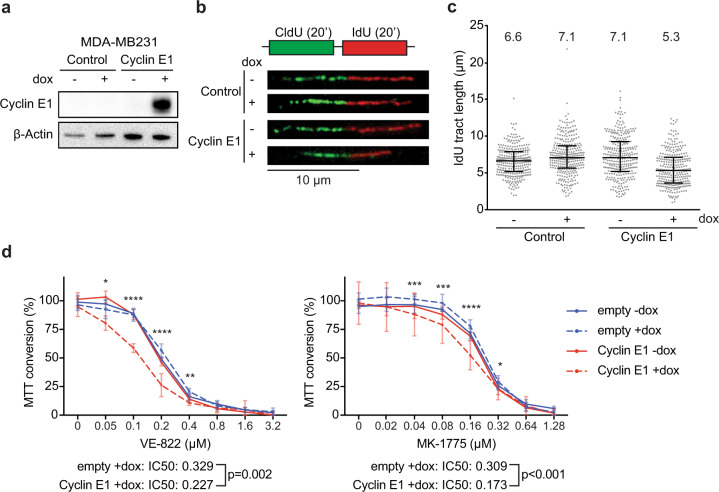
Overexpression of Cyclin E1 results in replication stress and increased sensitivity to ATR and WEE1 inhibition. **a** Immunoblotting of Cyclin E1 and β-Actin at 48 h after doxycycline addition to MDA-MB-231 cells. **b** Cells were treated with doxycycline as described for panel **a**. Subsequently, cells were pulse-labeled for 20 min with CldU (25 µM) and subsequently pulse-labeled for 20 min with IdU (250 µM). Representative DNA fibers are shown. Scale bar indicates 10 µm. **c** Quantification of IdU DNA fiber lengths as described in panel **b**. Per condition, 300 fibers were analyzed and individual datapoints and corresponding medians are shown. **d** MDA-MB-231 cell induced to express Cyclin E1 were treated for 4 days with ATR inhibitor (VE-822) in a range from 0 to 3.2 µM, or WEE1 inhibitor (MK-1775) in a range from 0 to 1.28 µM. Subsequently, MTT conversion was analyzed. Per experiment, six technical replicates per condition were included. Averages and standard deviations are plotted. Indicated *P* values were calculated to compare the relative MTT conversion between MDA-MB231 cells (Empty^+dox^ vs. Cyclin E1^+dox^) using two-tailed Student’s *t* test. *Indicates *P* < 0.05, ** indicates *P* < 0.01, *** indicates *P* < 0.001, **** indicates *P* < 0.0001. Mean IC50 values are indicated, and were statistically tested using a two-tailed Student’s *t* test. Additional comparisons between MDA-MB231 cells are provided in Supplementary Table 1.

To study the potential effects of Cyclin E1, encoded by the *CCNE1* gene, on DNA replication kinetics in vitro, we transduced MDA-MB-231 TNBC cells with a doxycycline-inducible Cyclin E1 construct (Fig. 1a). Cells were treated for 48 h with doxycycline to induce Cyclin E1 overexpression, and were then sequentially labeled with the thymidine analogues CldU and IdU to probe replication kinetics (Fig. 1b). Measurement of individual IdU tract lengths revealed that overexpression of Cyclin E1 resulted in a reduction in ongoing DNA synthesis speed of approximately 25% (Fig. 1c). To assess whether Cyclin E1 overexpression affects the sensitivity of cancer cells to inhibitors of cell cycle checkpoint kinases, we induced Cyclin E1 overexpression and inhibited ATR and WEE1 kinases using VE-822 and MK-1775 respectively (Fig. 1d, Supplementary Table 1). Induction of Cyclin E1 overexpression increased the sensitivity to ATR and WEE1 inhibitors in MDA-MB231 cells, as assessed using MTT assays (Fig. 1d). Taken together, overexpression of Cyclin E1 results in replication stress in TNBC cells and enhanced the sensitivity towards inhibitors of the WEE1 and ATR cell cycle checkpoint kinases.

### Analysis of breast cancer tissues

**Fig. 2:**
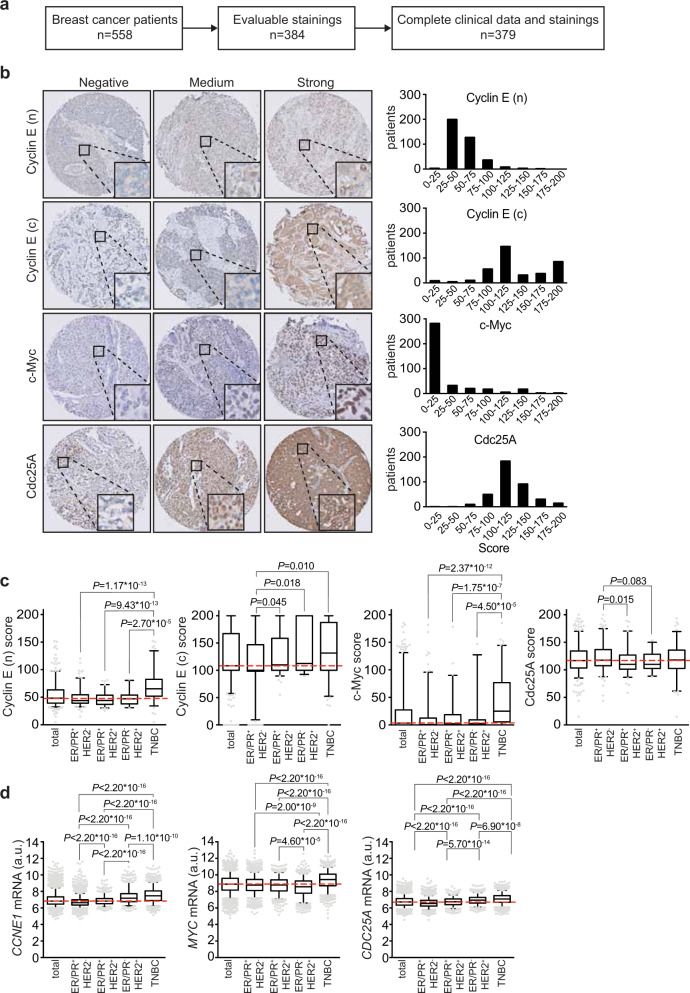
Analysis of immunohistochemical stainings and mRNA profiles in breast cancer patients. **a** Flow diagram indicating selection of patients included for immunohistochemical and clinicopathological analyses. **b** Analysis of staining intensities of oncogenes (*n* = 384), including cytoplasmic (c) and nuclear (n) Cyclin E, c-Myc, and Cdc25A. **c** Patients from the combined cohort (*n* = 384) and breast cancer subgroups ER/PR^+^HER2^−^ (*n* = 161), ER/PR^+^HER2^+^ (*n* = 90), ER/PR^−^HER2^+^ (*n* = 27) and TNBC (*n* = 106) were analyzed. Tumor tissue was immunohistochemically scored for expression of oncogenes (Cyclin E (n), Cyclin E (c), c-Myc, Cdc25A). Indicated *P* values were calculated using Mann–Whitney *U* test. **d** mRNA profiles of *CCNE1*, *CDC25A*, and *MYC* from breast cancer samples retrieved from the Gene Expression Omnibus (GEO, *n* = 7270). Breast cancer subgroups ER/PR^+^HER2^−^ (*n* = 4094), ER/PR^+^HER2^+^ (*n* = 812), ER/PR^−^HER2^+^ (*n* = 768) and TNBC (*n* = 1596) were analyzed. Indicated *P* values were calculated using Mann–Whitney *U* test. Box plots indicate medians and interquartile range, whiskers represent 10th and 90th percentile.

To further investigate oncogene-induced replication stress in clinical samples, we selected a study population that comprised 384 breast cancer patients (Fig. 2a), whose baseline clinical, pathological and treatment characteristics are summarized in Supplementary Table 2. Breast cancer patients were divided into four subgroups according to their hormone receptor status and HER2 expression. Molecular subgroup analysis showed that our cohort consisted of *n* = 161 ER/PR^+^HER2^−^, *n* = 90 ER/PR^+^HER2^+^, *n* = 27 ER/PR^−^HER2^+^, and *n* = 106 ER/PR^−^HER2^−^ (TNBC) patients (Supplementary Table 2). Compared to other patient subgroups, the median age at diagnosis was lowest for TNBC patients, followed by ER/R^−^HER2^+^ patients^23^ (Supplementary Table 2). In addition, tumor grade significantly varied across breast cancer subgroups (Supplementary Table 2, *P* = 1.72 × 10^−13^), and was highest in patients with TNBC (Supplementary Table 2, *P* < 0.05 for all tests). In accordance with treatment guidelines, chemotherapy was most frequently used in TNBC patients (66.0%), whereas radiotherapy and endocrine therapy were more frequently used in non-TNBC patients (Supplementary Table 3, *P* < 0.001 for all tests).

### Expression of Cyclin E, c-Myc, and Cdc25A in breast cancer subgroups

We next performed immunohistochemical analysis in breast cancer tissues taken prior to treatment (Supplementary Table 4) to examine the expression levels of Cyclin E (encoded by *CCNE1*) and c-Myc (encoded by *MYC*), two oncogenes that are frequently amplified in TNBC, and have been associated with replication stress in experimental models^24–27^ (Fig. 2b). For Cyclin E, we separately assessed nuclear and cytoplasmic Cyclin E (Fig. 2b), since cytoplasmic Cyclin E has been related to reduced breast cancer survival^28,29^. We also assessed expression of the Cdc25A phosphatase (Fig. 2b). Although Cdc25A is less frequently overexpressed in breast cancer^15^, Cdc25A overexpression is frequently used to induce replication stress in experimental models^30^, and has been linked to oncogenic activity^31,32^, and for that reason was included in our analysis.

Expression levels of nuclear Cyclin E were significantly higher in TNBC than in the other breast cancer subgroups (Supplementary Table 5, and Fig. 2c, *P* < 0.05 for all tests). In contrast, cytoplasmic Cyclin E levels were high in both TNBC and ER/PR^−^HER2^+^ tumors, compared to ER/PR^+^HER2^−^ tumors (Supplementary Table 5 and Fig. 2c, *P* < 0.05 for all tests). Expression levels of c-Myc were also higher in TNBC (Supplementary Table 5 and Fig. 2c, *P* < 0.001) compared to the other subgroups. Although TNBC tumors also displayed the highest levels of Cdc25A, these differences were not statistically significant (Supplementary Table 5 and Fig. 2c). We next analyzed mRNA expression levels of *CCNE1*, *MYC*, and *CDC25A* in a set of 7270 gene expression profiles from primary breast tumors obtained from the Gene Expression Omnibus (GEO)^33^. The mRNA expression of *CCNE1*, *MYC*, and to a lesser extend *CDC25A* were significantly higher in TNBC when compared to the other subgroups (Fig. 2d). These findings confirm at the mRNA level that, among breast cancer subgroups, TNBCs exhibited the highest expression levels of Cyclin E, c-Myc and Cdc25A.

### Levels of replication stress in breast cancer subgroups

**Fig. 3:**
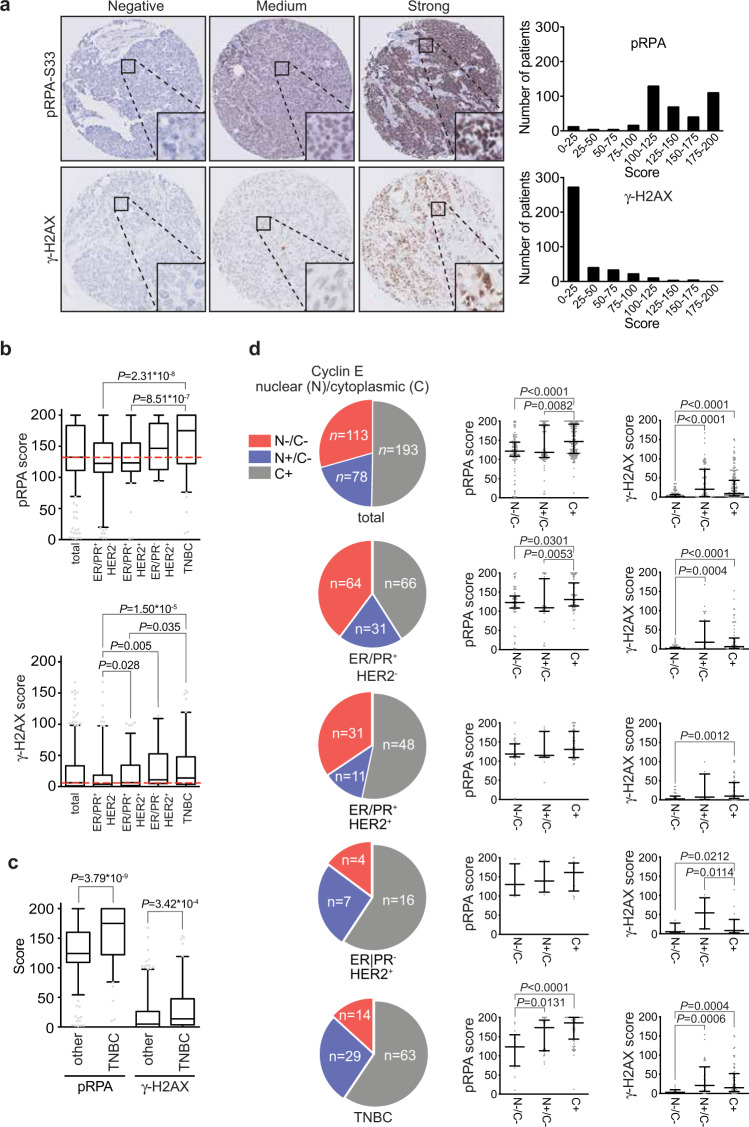
Expression of oncogenes and replication stress markers in breast cancer. **a** Analysis of staining intensities of replication stress markers, γ-H2AX and pRPA32 (Ser33) in breast cancer TMAs (*n* = 384). (**b**) Patients from the combined cohort (*n* = 384) and breast cancer subgroups ER/PR^+^HER2^−^ (*n* = 161), ER/PR^+^HER2^+^ (*n* = 90), ER/PR^−^HER2^+^ (*n* = 27) and ER/PR^−^HER2^−^ (*n* = 106) were analyzed. Tumor tissue was immunohistochemically scored for expression of oncogenes (Cyclin E (n), Cyclin E (c), c-Myc, Cdc25A), indicated *P* values were calculated using Mann–Whitney *U* test. Box plots represent medians and interquartile range. Whiskers represent 10th and 90th percentile. **c** TNBC patients (ER/PR^−^HER2^−^, *n* = 106) were compared with non-TNBC breast cancers (*n* = 278) for expression of replication stress markers pRPA and γ-H2AX. Box plots represent medians and interquartile range. Whiskers represent 10th and 90th percentile. **d** Tumor tissue from the combined cohort (*n* = 384) was immunohistochemically scored for expression of oncogenes (Cyclin E (n), Cyclin E (c), Cdc25A and c-Myc). Cyclin E scores were classified into nuclear and cytoplasm negative (N−/C−, *n* = 113), nuclear positive and cytoplasm negative (N+/C−, *n* = 78) and either nuclear positive or negative and cytoplasm positive (C+, *n* = 193). An additional subclassification was performed based on breast cancer subgroups ER/PR^+^HER2^−^, ER/PR^+^HER2^+^, ER/PR^−^HER2^+^ and TNBC. For all subgroups or the total cohort, tumor expression of replication stress markers (pRPA and γ-H2AX) was assessed, indicated *P* values were calculated using Mann–Whitney *U* test. “Ns” indicates not significant. * indicates *P* < 0.05, ** indicates *P* < 0.01, *** indicates *P* < 0.001, **** indicates *P* < 0.0001. Medians and standard deviations are indicated.

To determine levels of replication stress, we immunohistochemically examined the expression of phosphorylated RPA33 (further referred to as pRPA) in breast cancer tissues taken prior to treatment. In addition, we analyzed the expression of γ-H2AX, an established marker for collapsed replication forks and double-strand breaks, which are consequences of replication stress^34,35^. Representative immunohistochemical pRPA and γ-H2AX stainings are shown in Fig. 3a. We also compared expression of pRPA and γ-H2AX with other DNA damage response components in a subset of samples. Specifically, we immunohistochemically stained *n* = 45 cases for 53BP1 and FANCD2, two proteins involved in the repair of DNA lesions induced by replication stress (Supplementary Fig. 1a). High levels of 53BP1 were present in all cases analyzed (Supplementary Fig. 1b), whereas expression levels of FANCD2 showed larger variation (Supplementary Fig. 1b). Importantly, we found that expression of γ-H2AX and FANCD2 were associated (Supplementary Fig. 1c, *r* = 0.344, *P* = 0.028), in line with their roles in resolving the consequences of replication stress-induced DNA lesions. In contrast, 53BP1 expression did not show statistically significant associations with expression of either pRPA or γ-H2AX (Supplementary Fig. 1c).

Analysis of pRPA staining revealed that TNBC tumors displayed highest expression levels (Supplementary Table 5, Fig. 3b). Moreover, TNBC tumors showed higher pRPA levels than the combined non-TNBC tumors (Fig. 3c, *P* = 3.79 × 10^−9^). Also, γ-H2AX scores from TNBC tumors were significantly higher than ER/PR^+^/HER2^−^ and ER/PR^+^/HER2^+^ status (Supplementary Table 5, Fig. 3b, *P* = 1.50 × 10^−5^ and *P* = 0.035, respectively). Again, TNBC tumors displayed higher levels of γ-H2AX expression when compared to the combined group of non-TNBCs (Fig. 3c, *P* = 3.42 × 10^−^^4^). These data indicate that the expression of replication stress markers varies among breast cancer subgroups, with TNBCs and ER/PR^−^HER2^+^ tumors exhibiting highest levels.

Since the highest expression levels of c-Myc and Cyclin E as well as replication stress markers were observed in TNBC, we further analyzed relevant TNBC subgroups. Expression of the androgen receptor (AR) has been described to define a TNBC subgroup with distinct characteristics^36^. In our cohort, *n* = 29 out of 106 TNBC cases (27.4%) expressed the AR (Supplementary Fig. 2a, Supplementary Table 6a, b). We next analyzed the expression levels of replication stress markers in TNBC-AR^−^ and TNBC-AR^+^ subgroups (Supplementary Fig. 2b). Tumor expression of pRPA (Supplementary Fig. 2b, Supplementary Table 6a, *P* = 0.686) and γ-H2AX (Supplementary Fig. 2b, Supplementary Table 6a, *P* = 0.798) were not statistically different between the AR^+^ vs. AR^−^ TNBC cases. Likewise, tumor expression of oncogenes was similarly distributed in TNBC-AR^−^ and TNBC-AR^+^ subgroups (Supplementary Fig. 2b and Supplementary Table 6b, *P* > 0.05 for all tests). These data indicate that in this cohort, expression of replication stress markers and expression of oncogenes are similarly distributed in TNBC-AR^−^ and TNBC-AR^+^ subgroups.

### Correlations between replication stress markers and Cyclin E, c-Myc, and Cdc25A expression

To determine whether expression of Cyclin E, c-Myc, or Cdc25A was associated with replication stress in our study population, we examined associations between expression of Cdc25A, Cyclin E, and c-Myc with expression of replication stress markers pRPA and γ-H2AX. We first analyzed associations of replication stress markers with oncogene expression as continuous variables (Table 1), because no clear biphasic distributions of staining intensities were observed. Expression levels of pRPA were positively correlated with those of c-Myc (Table 1, *r* = 0.26, *P* < 0.001), as well as expression levels of nuclear Cyclin E (Table 1, *r* = 0.37, *P* < 0.001) and cytoplasmic Cyclin E (Table 1, *r* = 0.28, *P* < 0.001) in the entire cohort. Among breast cancer subgroups, the strongest correlations were found in TNBC between Cyclin E and pRPA expression (Table 1, *r* = 0.43, *P* < 0.001), and between c-Myc and pRPA (Table 1, *r* = 0.36, *P* < 0.001). Furthermore, Cyclin E expression was strongly correlated with levels of γ-H2AX staining (Table 1, *r* = 0.63, *P* < 0.001). Spearman correlation analysis of breast cancer subgroups revealed that the association between nuclear Cyclin E and γ-H2AX expression was strongest in ER/PR^−^HER2^+^ (Table 1, *r* = 0.86, *P* < 0.001) and TNBC (Table 1, *r* = 0.71, *P* < 0.001). Combined, these data indicate that expression of Cyclin E is associated with expression of replication stress markers in our study population, especially in the TNBC and ER/PR^−^HER2^+^ subgroups.

**Table 1:** Spearman rank correlation analysis of replication stress marker expression versus oncogene expression in the study population.

	BC Subgroup				
	Combined cohort	ER/PR^+^HER2^−^	ER/PR^+^HER2^+^	ER/PR^−^HER2^+^	TNBC
	*n* = 384	*n* = 161	*n* = 90	*n* = 27	*n* = 106
Variable	pRPA				
*Cdc25A*					
Correlation	−0.135	−0.269	−0.152	0.076	0.035
* P* value	0.008	0.001	0.153	0.707	0.719
*Cyclin E (n)*					
Correlation	0.371	0.090	0.289	0.232	0.432
* P* value	5.61 × 10^−14^	0.258	0.006	0.245	4.00 × 10^−6^
*Cyclin E (c)*					
Correlation	0.275	0.309	0.143	0.218	0.262
* P* value	4.25 × 10^−8^	6.50 × 10^−5^	0.180	0.274	0.007
*c-Myc*					
Correlation	0.256	0.214	0.180	0.133	0.359
* P* value	3.59 × 10^−7^	0.006	0.090	0.509	1.58 × 10^−4^
Variable	γ-H2AX				
*Cdc25A*					
Correlation	−0.030	−0.140	−0.037	0.368	0.170
* P* value	0.561	0.076	0.732	0.059	0.081
*Cyclin E (n)*					
Correlation	0.632	0.538	0.613	0.855	0.705
* P* value	3.07 × 10^−44^	1.80 × 10^−13^	1.34 × 10^−10^	1.40 × 10^–8^	3.53 × 10^−17^
*Cyclin E (c)*					
Correlation	0.192	0.209	0.258	−0.260	0.078
* P* value	1.55 × 10^−4^	0.008	0.014	0.190	0.424
*c-Myc*					
Correlation	0.129	0.113	0.032	0.214	−0.032
* P* value	0.011	0.153	0.763	0.283	0.743

Association analysis between oncogene expression and pRPA or γ-H2AX expression in the combined cohort (*n* = 384) and in breast cancer subgroups. Oncogene expression was used as a continuous variable in a Spearman rank correlation analysis.

Similar associations were observed when we dichotomized samples on the basis of oncogene expression. Specifically, Cyclin E stainings were categorized into nuclear and cytoplasmic negative (N−/C−, *n* = 113), nuclear positive and cytoplasmic negative (N+/C−, *n* = 78) and cytoplasmic positive with either nuclear positive or nuclear negative (C+, *n* = 193) (Table 2 and Fig. 3d). In line with our earlier results (Fig. 3b), we observed that highest pRPA and γ-H2AX expression levels were found in the TNBC subgroups with Cyclin E C+ (*n* = 63) and N+/C− (*n* = 29) as well as ER/PR^−^HER2^+^ subgroup with Cyclin E C+ (*n* = 16) and N+/C− (*n* = 7). In parallel, we dichotomized samples based on Cdc25A or c-MYC expression (Supplementary Fig. 3). High expression levels of Cdc25A did not show strong associations with increased levels of pRPA or γ-H2AX in breast cancer subgroups (Supplementary Fig. 3), whereas high expression of c-Myc was associated with increased levels of pRPA, predominantly in the TNBC subgroup (Supplementary Fig. 3).

**Table 2:** Regression analysis of replication stress marker expression versus oncogene expression in the study population.

BC subgroup	Mean	SD	P5	P50	P95
*γ-H2AX score*					
Cyclin E (N+/C−)					
Combined cohort (*n* = 113)	5.3	8.2	0	1.7	26.3
ER/PR^+^HER2^−^ (*n* = 64)	3.6	5.9	0	1	19.2
ER/PR^+^HER2^+^ (*n* = 31)	7.8	10.6	0	2.5	35
ER/PR^−^HER2^+^ (*n* = 4)	12.1	15.3	3.3	5	–
TNBC (*n* = 14)	5.2	7	0	2.9	–
Cyclin E (N+/C−)					
Combined cohort (*n* = 78)	39	42.3	0	20.8	112.7
ER/PR^+^HER2^−^ (*n* = 31)	37.4	44.8	0	17.5	133.8
ER/PR^+^HER2^+^ (*n* = 11)	30.8	36.3	0	7.5	–
ER/PR^−^HER2^+^ (*n* = 7)	59	37.8	10.8	54.2	–
TNBC (*n* = 29)	39.1	43.1	0	20.8	147.3
Cyclin E (C+)					
Combined cohort (*n* = 193)	27.2	34.8	0	9.2	102.9
ER/PR^+^HER2^−^ (*n* = 66)	23.9	35.4	0	6.3	104
ER/PR^+^HER2^+^ (*n* = 48)	26.5	30	0	10	99.9
ER/PR^−^HER2^+^ (*n* = 16)	22.6	32.3	0	7.9	–
TNBC (*n* = 63)	32.4	38.1	0	15	119.5
*pRPA score*					
Cyclin E (N−/C−)					
Combined cohort (*n* = 113)	120.2	44.5	8	121.7	192.9
ER/PR^+^HER2^−^ (*n* = 64)	119.4	45.8	6.3	122.9	195.9
ER/PR^+^HER2^+^ (*n* = 31)	121	39.6	2.2	118.3	182
ER/PR^−^HER2^+^ (*n* = 4)	138.8	43.6	98.3	130	–
TNBC (*n* = 14)	116.9	52.5	10	123.3	–
Cyclin E (N+/C−)					
Combined cohort (*n* = 78)	137	49.3	42.8	118.3	200
ER/PR^+^HER2^−^ (*n* = 31)	120	54.1	10	109.2	200
ER/PR^+^HER2^+^ (*n* = 11)	126.5	42.6	44.2	115	–
ER/PR^−^HER2^+^ (*n* = 7)	146.7	38.1	103.3	139.2	–
TNBC (*n* = 29)	156.9	42.3	81.3	173.3	200
Cyclin E (C+)					
Combined cohort (*n* = 193)	151	38.8	100.6	147	200
ER/PR^+^HER2^−^ (*n* = 66)	140.7	36.7	96.5	130.4	200
ER/PR^+^HER2^+^ (*n* = 48)	140.5	34.4	97.5	130.4	200
ER/PR^−^HER2^+^ (*n* = 16)	150.5	39.7	92.5	161.3	–

Association analysis between oncogene expression and pRPA or γ-H2AX expression in the combined cohort (*n* = 384) and in breast cancer subgroups.

### AR status vs. expression of oncogenes and markers of replication stress

To test whether the association between oncogene expression and expression of replication stress markers was affected by AR expression in TNBCs, Spearman correlation analyses were performed. A stronger association between pRPA and Cyclin E was observed in AR-negative TNBCs (Supplementary Table 7a, *r* = 0.463, *P* < 0.001), when compared to AR-positive TNBCs (Supplementary Table 7a, *r* = 0.327, *P* = 0.083). Similarly, Spearman correlation analysis underscored that the association between Cyclin E and γ-H2AX was stronger in AR-negative tumors (Supplementary Table 7b, *r* = 0.755, *P* < 0.05) than in AR-positive tumors (Supplementary Table 7b, *r* = 0.574, *P* = 0.001). In line with this observation, Cdc25A and Cyclin E showed weaker associations in AR-positive tumors (Supplementary Table 7a, Cdc25A: *r* = −0.214, *P* = 0.265; Cyclin E: *r* = 0.092, *P* = 0.637), than in AR-negative tumors (Supplementary Table 7a, Cdc25A: *r* = 0.131, *P* = 0.256; Cyclin E: *r* = 0.320, *P* = 0.005). In addition, AR-negative tumors showed stronger associations between γ-H2AX and Cdc25A (Supplementary Table 7b, *r* = 0.275, *P* = 0.016) and between γ-H2AX and Cyclin E (Supplementary Table 7b, *r* = 0.118, *P* = 0.306) than AR-positive tumors (Supplementary Table 7b, Cdc25A: *r* = −0.100, *P* = 0.607 and (Cyclin E: *r* = 0.048, *P* = 0.806). In conclusion, markers of replication stress appear equally expressed in AR-negative and AR-positive TNBCs, although the associations between replication stress (pRPA, γ-H2AX) and oncogene expression (Cdc25A, Cyclin E) are strongest in AR-negative TNBCs within our cohort.

### Associations of expression of replication stress markers with clinicopathological characteristics and tumor expression of Cyclin E, c-Myc and Cdc25A

Linear regression analyses were performed to evaluate the relation between expression of replication stress markers versus clinicopathological characteristics and tumor expression of Cdc25A, Cyclin E, and c-Myc. Univariate regression analysis showed that pRPA was associated with γ-H2AX (Table 3, *β* = 0.409, *P* < 0.001). Also, pRPA was associated with positivity for cytoplasmic Cyclin E (Table 3, *β* = 0.345, *P* < 0.001). In contrast, weaker associations were found between oncogene expression and pRPA levels (Table 3). The covariates from univariate regression analyses that displayed *P* < 0.05 were included for multivariate analysis, and showed that pRPA was weakly related to γ-H2AX (Table 3, *β* = 0.351, *P* < 0.001). Conversely, tumor expression of γ-H2AX was associated with nuclear Cyclin E (Table 3, *β* = 0.407, *P* < 0.001) and with cytoplasmic Cyclin E (Table 3, *β* = 0.324, *P* < 0.001). No strong independent associations were found between clinicopathological parameters and pRPA or γ-H2AX expression. In summary, our findings indicate that replication stress measured with pRPA, corrected for age, tumor subgroup, tumor grade, and tumor expression, is related to γ-H2AX, whereas replication stress quantified with γ-H2AX, corrected for tumor subgroup and tumor expression, is positively associated with tumor expression of nuclear and cytoplasmic Cyclin E.

**Table 3:** Relation between replication stress markers versus oncogene expression and clinicopathological characteristics of the study population.

	Univariate (variable: pRPA)			Multivariate (variable: pRPA)		
	Beta	95% CI	*P* value	Beta	95% CI	*P* value
Age (continuous)	−0.125	−0.684 to [−0.073]	0.015	−0.058	−0.459 to 0.106	0.219
Tumor subgroups			2.44 × 10^−7^		6.97 × 10^−22^	
ER/PR^+^HER2^−^	*Ref*.			*Ref*.		
ER/PR^+^HER2^+^	0.037	−7.293 to 14.989	0.497	0.019	−8.136 to 12.126	0.699
ER/PR^−^HER2^−^	0.105	0.755–36.488	0.041	0.077	−2.761 to 29.806	0.103
TNBC	0.304	19.817–41.120	3.64 × 10^−8^	0.198	8.872 to 30.720	4.14 × 10^−4^
Tumor stage			0.311			
I	*Ref*.					
II	0.042	−5.638 to 13.349	0.425			
III	−0.059	−29.687 to 8.204	0.266			
Tumor grade			0.006			
I	*Ref*.			*Ref*.		
II	0.001	−12.632 to 12.902	0.983	−0.019	−12.979 to 9.440	0.756
III	0.165	2.439–27.318	0.019	−0.083	−19.799 to 4.908	0.237
CDC25A	−0.055	−0.273 to 0.080	0.284			
Cyclin E subgroups			2.66 × 10^−8^			
N−/C−	*Ref*.			*Ref*.		
N+/C−	0.155	4.729–29.564	0.007	−0.031	−15.607 to 8.765	0.581
C+	0.345	20.763–40.844	3.87 × 10^−9^	0.152	3.876 to 23.371	0.006
c-MYC	0.248	0.167–0.386	1.00 × 10^−6^	0.154	0.059 to 0.284	0.003
γ-H2AX	0.409	0.421–0.667	1.03 × 10^−16^	0.373	0.371–0.621	5.57 × 10^−14^

	Univariate (variable: γ-H2AX)			Multivariate (variable: γ-H2AX)		
Age (continuous)	8.56 × 10^−4^	−0.229 to 0.233	0.987			
Tumor subgroups		0.025			1.11*10^−21^	
ER/PR^+^HER2^−^	*Ref*.			*Ref*.		
ER/PR^+^HER2^+^	0.024	−6.731 to 10.561	0.663	0.017	−6.299 to 8.930	0.734
ER/PR^−^HER2^−^	0.065	−5.284 to 22.448	0.224	−0.008	−13.327 to 11.262	0.869
TNBC	0.163	4.014–20.547	0.004	−0.014	−8.688 to 6.639	0.793
Tumor stage			0.241			
I	*Ref*.					
II	0.085	−1.265 to 12.995	0.107			
III	0.043	−8.351 to 20.105	0.417			
Tumor grade			0.058			
I	*Ref*.					
II	0.007	−9.175 to 10.132	0.922			
III	0.127	−0.799 to 18.013	0.073			
CDC25A	0.019	−0.108 to 0.158	0.711			
Cyclin E subgroups			1.02 × 10^−12^			
N−/C−	*Ref*.			*Ref*.		
N+/C−	0.407	24.693–42.859	1.60 × 10^−12^	0.355	20.800–38.243	1.01*10^−10^
C+	0.324	14.414–29.103	1.23 × 10^−8^	0.201	6.250–20.784	2.92*10^−4^
c-MYC	0.026	−0.063 to 0.107	0.612			
pRPA	0.409	0.238–0.377	1.03 × 10^−16^	0.368	0.205–0.348	2.17*10^−13^

(a) Univariate linear regression analyses were performed to analyze the relation between expression of the replication stress markers pRPA or γ-H2AX vs. clinicopathological characteristics and tumor expression of Cyclin E, c-Myc, or Cdc25A (*n* = 379). Comparisons with a *P* value < 0.05 in univariate linear regression analyses were selected for multivariate linear regression analyses.

### Associations between expression of oncogenes and markers of replication stress with survival

We next analyzed the relationship between the expression of Cyclin E, c-MYC, Cdc25A, and markers of replication stress with disease-free survival (DFS), recurrence-free survival (RFS) or overall survival (OS) in our breast cancer cohort (n = 379) using Cox regression analyses (Table 4). Univariate analyses showed that positivity of nuclear Cyclin E expression was associated with worse OS (Table 4, *β* = 0.633, *P* = 0.035) and borderline associated with DFS (Table 4, *β* = 0.565, *P* = 0.058) but not RFS (Table 4, *β* = 0.162, *P* = 0.619). Concerning MYC expression, high expression was also associated with poor OS (Table 4, *β* = 0.535, *P* = 0.023) and DFS (Table 4, *β* = 0.555, *P* = 0.019). For multivariate Cox regression analyses, age, tumor size, tumor grade, lymph node involvement, ER status, HER2 status, and treatment regimen were included (Table 4) and were not associated with expression of Cyclin E, Cdc25A, c-Myc or γ-H2AX.

**Table 4:** Associations between tumor expression and survival.

	Univariate (OS)			Multivariate (OS)		
	Beta	95% CI	*P-*value	Beta	95% CI	*P-*value
Cyclin E						
N−/C−	*Ref*.			*Ref*.		
N+/C−	0.633	1.046–3.391	0.035	0.267	0.677–2.519	0.425
C+	0.219	0.730–2.123	0.422	0.046	0.571–1.921	0.881
Cdc25A						
Low	*Ref*.			*Ref*.		
High	0.203	0.790–1.898	0.364	0.106	0.692–1.785	0.662
c-Myc						
Low	*Ref*.			*Ref*.		
High	0.535	1.076–2.712	0.023	0.098	0.632–1.926	0.73
pRPA						
Low	*Ref*.			*Ref*.		
High	0.131	0.735–1.770	0.558	0.255	0.799–2.085	0.297
γ-H2AX						
Low	*Ref*.			*Ref*.		
High	0.333	0.890–2.186	0.146	0.284	0.826–2.137	0.241

	Univariate (DFS)			Multivariate (DFS)		
Cyclin E						
N−/C−	*Ref*.			*Ref*.		
N+/C−	0.565	0.980–3.162	0.058	0.191	0.626–2.340	0.57
C+	0.127	0.667–1.933	0.64	−0.043	0.525–1.749	0.889
Cdc25A						
Low	*Ref*.			*Ref*.		
High	0.102	0.715–1.716	0.647	−0.024	0.609–1.563	0.92
c-Myc						
Low	*Ref*.			*Ref*.		
High	0.555	1.097–2.765	0.019	0.162	0.676–2.043	0.566
pRPA						
Low	*Ref*.			*Ref*.		
High	0.108	0.719–1.726	0.629	0.09	0.682–1.755	0.708
γ-H2AX						
Low	*Ref*.			*Ref*.		
High	0.252	0.827–2.001	0.263	0.119	0.707–1.797	0.616

	Univariate (RFS)			Multivariate (RFS)		
Cyclin E						
N−/C−	*Ref*.			*Ref*.		
N+/C−	0.162	0.621–2.227	0.619	0.083	0.544–2.173	0.813
C+	0.124	0.673–1.902	0.641	−0.094	0.508–1.630	0.752
Cdc25A						
Low	*Ref*.			*Ref*.		
High	−0.217	0.509–1.274	0.355	−0.212	0.500–1.309	0.388
c-Myc						
Low	*Ref*.			*Ref*.		
High	0.419	0.955–2.419	0.077	−0.085	0.522–1.616	0.768
pRPA						
Low	*Ref*.			*Ref*.		
High	0.127	0.723–1.780	0.582	−0.081	0.568–1.497	0.743
γ-H2AX						
Low	*Ref*.			*Ref*.		
High	0.05	0.671–1.649	0.826	0.04	0.648–1.670	0.870

Univariate Cox regression analyses were performed to analyze the relation between tumor expression of Cyclin E, c-Myc, Cdc25A, pRPA, or γ-H2AX on overall survival (OS), disease-free survival (DFS) and recurrence-free survival (RFS) on ER/PR^+^HER2^−^ (*n* = 159), ER/PR^+^HER2^+^ (*n* = 90), ER/PR^−^HER2^+^ (*n* = 26) and TNBC (*n* = 104) breast cancer patients. For multivariate Cox regression analyses, age, tumor size, tumor grade, lymph node involvement, ER status, HER2 status, and treatment regimen were used as covariates.

Next, we analyzed DFS on the basis of those publicly available patient data retrieved from GEO, of which also clinical data was available (*n* = 3450 out of *n* = 7270). A shorter DFS was found in patients with ER^+^/HER2^+^ (*n* = 341) and ER^−^/HER2^+^ (*n* = 291) tumors, when compared to other subgroups (Supplementary Fig. 4a), and a shorter OS in the TNBC (*n* = 263) and ER^−^/HER2^+^ (*n* = 341) patients (Supplementary Fig. 4b). When we evaluated the relation between *CCNE1* mRNA expression and DFS (*n* = 846) or OS (*n* = 632) using multivariate Cox regression analysis of data from publicly available mRNA samples of primary breast tumors, we found no associations between *CCNE1* mRNA expression and DFS. In contrast, a higher mRNA expression level of *CCNE1* was associated with reduced OS (Supplementary Table 8, HR = 1.660, *P* = 0.004) in the ER^+^/HER2^−^ subgroup (*n* = 417).

## Discussion

In the present study, we examined the relation between oncogene expression and replication stress marker expression in breast cancer subgroups. Our immunohistochemical analyses show that levels of replication stress and oncogene expression vary among breast cancer subgroups and that the highest expression levels of replication stress markers and oncogenes were found in the TNBC and ER/PR^−^HER2^+^ subgroups. Furthermore, both nuclear and cytoplasmic Cyclin E expression, and to a lesser extend c-Myc expression, were strongly associated with the levels of replication stress. These findings are relevant in the context of ongoing clinical studies using novel agents that target replication stress, for which proper patient selection is warranted.

Previous immunohistochemical studies showed that Cdc25A was highly expressed in 69.6% of human breast carcinomas analyzed (*n* = 46)^37^. A second study revealed that Cdc25A was overexpressed in 47% of breast cancer cases, in a cohort of 144 patients^15^. In comparison, in the present study 29.5% of tumors displayed strong Cdc25A staining intensity. Cdc25A has also been shown to be overexpressed in other cancer types, including high-grade serous ovarian cancer and head-and-neck squamous cell carcinoma^31,38^, which are characterized by genomic instability^39,40^.

Concerning c-Myc expression, our results indicated that high c-Myc expression was predominantly observed in TNBCs, and that c-Myc expression was associated with expression of the replication stress marker pRPA. These findings are in line with previous reports, showing frequent *MYC* amplification in TNBC^24^. Importantly, our results provide confirmation that the link between c-Myc-overexpression and induction of replication stress is also observed in patient samples^13,26,41^.

*CCNE1* is frequently amplified in TNBC, in line with our finding that high levels of Cyclin E expression is most prominent in TNBC cases^24^. Importantly, our observation that Cyclin E expression is associated with expression of replication stress markers is in line with experimental models, in which Cyclin E overexpression has been shown to trigger a DNA damage response^12,14,42^. Specific isoforms of Cyclin E, so-called low molecular-weight Cyclin E isoforms (LMW-E) are suggested to accumulate in the cytoplasm because they lack the NH_2_-terminal nuclear localization signal^43^. In line with experiments in which expression of cytoplasmic Cyclin E was shown to induce various features that relate to replication stress, including chromosome missegregation^44^, our data show that expression of cytoplasmic Cyclin E, like expression of nuclear Cyclin E, is associated with expression of replication stress markers pRPA and γ-H2AX. Of note, we found that cytoplasmic and nuclear Cyclin E showed a similar distribution among breast cancer subgroups, with highest expression observed in TNBC. However, no clear biphasic staining distributions of nuclear or cytoplasmic Cyclin E were observed. For this reason, we analyzed the expression of Cyclin E and other markers as continuous variables.

Survival analysis of our cohort of patients and publicly available patient data showed that higher levels of replication stress (pRPA) or high Cyclin E expression levels were associated with worse DFS, whereas a higher nuclear Cyclin E levels was related with worse OS, albeit only in univariate analysis. *CCNE1* mRNA was not found to be independently associated with DFS or OS in public data, except in the ER^+^/HER2^−^ subgroup, in which higher levels of *CCNE1* expression were associated with worse OS. Immunohistochemical analysis of Cyclin E previously identified Cyclin E as an independent predictor of survival^45^, although hormone receptor status was not included in this analysis. Also, total and low-molecular-weight Cyclin E levels, as assessed by Western blot, were shown to be independent predictors of overall survival in a breast cancer cohort^46^. Studies in other cancer types, including serous ovarian cancers and endometrial carcinomas, also showed that *CCNE1* amplification or Cyclin E overexpression was associated with more aggressive tumor features, but was not an independent predictor factor of survival^47–49^. However, high Cyclin E expression was shown to be a significant predictive marker for survival in suboptimally debulked ovarian cancers^50^.

Taken together, our findings indicate that among breast cancer subgroups, TNBCs and ER/PR^−^HER2^+^ tumors are characterized by overexpression of the c-Myc and Cyclin E oncogenes, and by higher expression levels of replication stress markers. These findings are relevant, as increasing numbers of drugs are being developed that target cancer cells with high levels of replication stress. Specifically, inhibitors of the cell cycle checkpoint kinases Chk1 and ATR are currently being tested in combination with genotoxic agents that interfere with DNA replication^51,52^. In parallel, inhibitors of the WEE1 kinase have been developed. The potential of WEE1 inhibition was early on attributed to high levels of replication stress^53^ and preclinical data indicated that WEE1 inhibition would be preferentially effective in Cyclin E-overexpressing cancer cells^54^. In line with these data, ovarian cancer patients that responded favorably to WEE1 inhibitor treatment more frequently showed tumor overexpression of Cyclin E^55^. Based on these observations, a clinical trial testing WEE1 inhibitor treatment in patients selected on *CCNE1* amplification is currently ongoing (clinicaltrials.gov identifier: NCT03253679).

Although different cell cycle checkpoint inhibitors are already in clinical development, an effective patient selection strategy is required to identify those patients who might benefit from these drugs. For breast cancer patients, our data underscore that overexpression of nuclear and/or cytoplasmic Cyclin E could be used as a selection criterion for treatment with drugs that target replication stress, including inhibitors of WEE1 and ATR.

## Methods

### Cell lines

TNBC cell lines MDA-MB-231 were obtained from ATCC (#HTB26) and were maintained in Dulbecco’s Minimum Essential Media (DMEM, Thermofisher), supplemented with 10% (v/v), fetal calf serum and 1% penicillin/streptomycin (Gibco). MDA-MB-231 cells were grown at 37 °C in normoxic conditions (20% oxygen and 5% CO_2_).

### DNA cloning and retroviral transduction

MDA-MB-231 cells were engineered to express Cyclin E1 in a doxycycline-inducible manner. First, cells were stably transduced with pRetroX-Tet-On Advanced (Clontech). To this end, HEK-293T cells were transfected with 10 µg of pRetroX-Tet-On Advanced, 2.5 µg of pMDg and 7.5 µg of pMD-g/p as described previously^56^. At 24, 36, and 48 h after transfection, virus-containing supernatant was filtered and added to target cells, which were subsequently selected for 7 days using geneticin (G418 Sulfate, 800 µg/mL, Thermofisher). Next, MDA-MB-231 cells harboring pRetroX-Tet-On Advanced were transduced with pRetroX-Tight-Pur containing *CCNE1*. To this end, Human *CCNE1* was PCR amplified from Rc-CycE, which was a gift from Bob Weinberg (Plasmid #8963, Addgene)^57^ using the following oligos: forward: 5′-CGCGGCCGCCATGAAGGAGGACGGCGGCGCG-3′, reverse: 5′-GATGAATTCTCACGCCATTTCCGGCCC-3′. The resulting fragments were cloned into pJET1.2/blunt, GeneJET, (ThermoFisher). *CCNE1* was subcloned into pRetroX-Tight-Pur using NotI and EcoRI restriction sites. Subsequently, cell lines harboring pRetroX-Tet-On Advanced were transduced with pRetroX-Tight-Pur containing *CCNE1* or empty plasmid. After 3 rounds of transduction, MDA-MB-231 cells were selected for 2 days with 1 µg/mL of puromycin dihydrochloride (Sigma).

### Western blotting

MDA-MB-231 cells were lysed in M-PER lysis buffer (Pierce), supplemented with protease and phosphatase inhibitor cocktail (Thermo Scientific). Protein content was measured using the Pierce BCA protein quantification Kit (Thermo Scientific). Protein samples were separated using sodium dodecyl sulfate-polyacrylamide gels (SDS-PAGE) and transferred to polyvinylidene fluoride membranes (Immobilon). Membranes were blocked in 5% skimmed milk (Sigma), in tris-buffered saline (TBS) containing 0.05% Tween-20 (Sigma) and incubated overnight with primary antibodies at 4 °C and subsequently incubated with secondary antibodies for 1 h at room temperature. Primary antibodies used were mouse anti-Cyclin E1 (Abcam, ab3927, 1:500) and mouse anti-β-actin (MpBiomedicals, 69100, 1:10,000). Secondary antibodies used were horseradish peroxidase-linked anti-mouse IgG (1:2000, DAKO) and visualized using chemiluminescence (Lumi-Light, Roche Diagnostics) on a Bio-Rad bioluminescence device. Protein imaging was performed using Image Lab software (Bio-Rad). All blots derive from the same experiment and were processed in parallel.

### DNA fiber analysis

To assess replication dynamics, MDA-MB-231 doxycycline-inducible cell lines were pulse-labeled with CldU (25 µM) for 20 min. Next, cells were washed with warm medium and pulse-labeled with IdU (250 µM) for 20 min. Cells were collected using trypsin and lysed on top of a microscope slide in lysis solution (0.5% SDS, 200 mM Tris [pH 7.4], 50 mM EDTA). DNA fibers were spread by tilting the microscope slide and were subsequently air-dried and fixed in methanol/acetic acid (3:1) for 10 min. Next, DNA spreads were immersed in 2.5 M HCl for 75 min. DNA fibers were blocked in blocking solution (5% bovine serum albumin in PBS) for 30 min and incubated with primary antibodies for 60 min at room temperature. CldU was detected with rat anti-BrdU (1:1000, Abcam, ab6326), whereas IdU was detected with mouse anti-BrdU (1:250, BD Biosciences, Clone B44). Secondary antibodies used for detection were Alexa488-conjugated goat anti-rat IgG (1:500) and Alexa647-conjugated goat anti-mouse IgG (1:500). Images were acquired on a Leica DM-6000B (63× immersion objective with 1.30 NA) fluorescence microscope, equipped with Leica Application Suite software. The lengths of 300 IdU tracts were measured per condition using ImageJ software.

### MTT assays

MDA-MB-231 cell lines harboring doxycycline-inducible Cyclin E1 were left untreated or treated with doxycycline (1 µg/ml) for 48 h. Subsequently, cells were re-plated in 96-wells at 1000 cells per well in the continued presence or absence of doxycycline, and allowed to attach for 24 h. ATR inhibitor VE-822 (Axon) or Wee1 inhibitor MK1775 (Axon) was added at indicated concentrations for 4 days. Next, cells were incubated with methylthiazol tetrazolium (MTT, final concentration 0.5 mg/ml) for 4 h. After removal of medium, formazan crystals were dissolved in dimethyl sulfoxide (DMSO). Absorbance was measured at 520 nm, and was quantified using a Benchmark III spectrophotometer (Bio-Rad). MTT conversion was plotted relative to the untreated cells. Per experiment, six replicates per condition were included. IC50 values were calculated using logistic regression model with the ATT Bioquest IC50 tool, with the minimum response set to zero. A two-sided Students *t* test was used to compare IC50 values.

### Breast cancer tissue

Immunohistochemical analysis was performed on tissue specimens taken prior to treatment of 558 patients with breast cancer who underwent surgery at the University Medical Center Groningen (UMCG). Tissue collection and storage of clinicopathological and follow up data was only performed upon patients’ approval via informed consent. Clinical data was collected in the UMCG and stored digitally in a central database, which is solely accessible by two dedicated data managers. Statistical analysis was performed with an anonymized dataset extracted from the central database. Protection of patient identity was thereby warranted and according to Dutch law no further Institutional Review Board approval was necessary. A first cohort (cohort A) consisted of 450 consecutive patients, of whom tumor specimens were collected between 1996 and 2005, considering the availability of sufficient paraffin-embedded tissue. A second cohort (cohort B) consisted of 108 consecutive patients with TNBC, of whom tumor specimens were collected from 2005 to 2010. Clinicopathological data gathered in this study was anonymized, and used in accordance with the Declaration of Helsinki, and in line with the regulations posed by the Institutional Review Board (IRB) of the UMCG. Tumor specimens were processed to generate nine tissue microarrays (TMAs). The first seven TMAs contained samples from cohort A, as described previously^58,59^. The final two TMAs contained TNBC cases from cohort B. For each tumor, three different cores were included in TMAs in a random fashion.

### Immunohistochemistry

TMA slices were deparaffinized using xylene. Slides were incubated for 30 min in 0.3% hydrogen peroxidase (H_2_O_2_) to suppress endogenous peroxidase activity. Immunohistochemistry was performed with primary antibodies against Cdc25A (1:400; rabbit, #sc-97, clone 144; Santa Cruz Biotechnology, CA, USA), Cyclin E (1:1000; rabbit, #sc-198, clone C19; Santa Cruz Biotechnology, CA, USA), c-Myc (RTU; rabbit, #790–4628, clone Y69; Roche, Basel, Switzerland), phospho-RPA32 (Ser33) (1:6400; rabbit, #A300-246A, clone S33; Bethyl, TX, USA), γ-H2AX (1:300; mouse, #05–636, clone JBW301; Millipore, Amsterdam, The Netherlands), the AR (RTU; rabbit, #760–4605, clone SP107; Roche, Basel, Switzerland), 53BP1 (1:300; rabbit, #IHC-00001; Bethyl, TX, USA) and FANCD2 (1:400; rabbit, #IHC-00624; Bethyl, TX, USA). Staining was detected by the application of 3,3-diaminobenzidine (DAB), and hematoxylin as a counterstaining. For c-Myc and pRPA, the complete staining procedure was performed on an autostainer (BenchMark Ultra IHC/ISH, Roche, Basel, Switzerland). Additional information about antibodies and staining protocols is provided in Supplemental Table 4.

Scoring was performed semi-quantitatively by two independent researchers, without knowledge of clinical data, and was supervised by a breast cancer pathologist. Stainings were categorized according to percentages of cells that showed staining and on intensity of staining. Staining intensity was scored in three categories: 0 (negative), 1 (medium), and 2 (high). In order to calculate the score for each core, the percentage of cells in each group was multiplied by their intensity score, resulting in a range from 0 to 200 points. Next, the scores from each case and staining were averaged and considered for analysis.

For Cdc25A, only nuclear staining was considered, in line with a previous study^60^. For Cyclin E, nuclear and cytoplasmic staining were scored individually^28^. In addition, nuclear c-Myc, pRPA, γ-H2AX, 53BP1, and FANCD2 stainings were evaluated. A concordance of more than 90% was found between observers. Discordant scores were reviewed and adjusted to consensus. The status of ER, PR, HER2, and AR was determined according to the guidelines of the American Society of Clinical Oncology/College of American Pathologists by counting at least 100 cells.

Immunohistochemical stainings were considered evaluable when a tumor core contained at least 10% tumor cells. In addition, tumor stainings were included for analysis when at least 2 out of 3 cores were evaluable. Core loss over 558 cases was on average 15.1% (Cdc25A, *n* = 106 [18.9%]; nuclear Cyclin E, *n* = 74 [13.2%]; cytoplasmic Cyclin E, *n* = 82 [14.6%]; c-Myc, *n* = 68 [12.1%]; pRPA, *n* = 77 [13.8%]; and γ-H2AX, *n* = 99 [17.7%]). For 384 out of 558 cases, all five stainings (pRPA, γ-H2AX, Cyclin E, c-Myc and Cdc25A) were evaluable. Of 384 cases with complete evaluable immunohistochemical stainings, 379 had complete clinicopathological data available, and were included for statistical analysis.

### Evaluation of mRNA expression of CCNE1, MYC and CDC25A

Publicly available mRNA profiles of 7270 primary breast tumors were collected from GEO platforms GPL96 (generated with Affymetrix HG-U133A) and GPL570 (generated with Affymetrix HG-U133 Plus 2.0) as previously described^33^. Expression profiles were batch corrected using COMBAT^61^. *CCNE1* expression values were calculated using probe 213523_at. *CDC25A* expression values were calculated using probe 204695_at. *MYC* expression values were calculated using probe 202431_s_at.

### Statistical analyses

Analyses were performed on the total study population as well as on four patient subgroups based on hormone receptor status and HER2 expression. Differences regarding clinicopathological features, treatment, and immunohistochemical expression levels between the four groups were analyzed using Pearson chi-square tests in case of categorical variables, while Kruskal–Wallis tests and Mann–Whitney *U* tests were used in case of continuous variables.

Univariate linear regression analyses were performed to study the relation between expression of replication stress markers versus clinicopathological characteristics and tumor expression of Cyclin E, c-Myc, or Cdc25A. Comparisons that reached *P* < 0.05 in univariate linear regression analyses were selected for multivariate linear regression analyses. All statistical analyses in this study were performed using SPSS Statistics 23.0 (IBM).

Associations between mRNA expression levels and survival in breast cancer subgroups were determined using multivariate Cox regression analyses with age, tumor size, tumor grade, lymph node involvement, ER status, HER2 status, and treatment regimen as co-variates. DFS was calculated as the interval between date of diagnosis to date of diagnosis of distant metastasis. RFS was based on the interval between date of diagnosis of disease to date of diagnosis of DM or date of overall death. OS was calculated as the interval between date of diagnosis to date of death by any cause. Survival probabilities for different breast cancer subgroups were calculated using Kaplan–Meier curves.

### Reporting summary

Further information on experimental design is available in the Nature Research Reporting Summary linked to this paper.

## Supplementary information

Supplemental Information

Reporting Summary

## Data Availability

The datasets generated and analyzed during the current study are publicly available in Mendeley Data: 10.17632/4pnhd8d7rw.1^62^. A metadata record describing these datasets is available in figshare: 10.6084/m9.figshare.12657233^63^. Uncropped blots from Fig. 1, are available as part of the Supplementary information files (Supplementary Fig. 5).
