# Supplementary material for: Cyclin E expression is associated with high levels of replication stress in triple-negative breast cancer
Source: NPJ Breast Cancer. 2020 Sep 7;6:40. doi: 10.1038/s41523-020-00181-w (PMC7477160; doi:10.1038/s41523-020-00181-w)
Supplement: Supplementary file 1 — Supplemental Information [file 41523_2020_181_MOESM1_ESM.pdf]

Supplemental Information

Supplemental Fig. 1: Expression of DNA damage response markers FANCD2 and 53bp1 in breast cancer samples.

(a) Representative stainings of 53BP1 and FANCD2 in TNBC patient samples. **(b)** Analysis of staining intensities of pRPA (n=48), γ-H2AX (n=43), 53BP1 (n=45) and FANCD2 (n=45). **(c)** Spearman correlation analysis of expression of 53BP1 and FANCD2 versus pRPA (n=45) and γ-H2AX (n=41) in a subset of breast cancer cases.

Supplemental Fig. 2: Androgen receptor expression in triple-negative breast cancers.
(a) Representative immunohistochemical stainings of the androgen receptor in TNBC patients ($ER/PR^{-}HER2^{-}$, n=106). **(b)** TNBC patients were subclassified based on the presence or absence of AR staining. $TNBC\text{-}AR^{-}$ (n=77) and $TNBC\text{-}AR^{+}$ (n=29) were compared based on their scores on replication stress markers and oncogenes.

Supplemental Fig. 3: Tumor expression of replication stress markers in breast cancer.
Tumor tissue from the combined cohort (n=384) was immunohistochemically scored for expression of oncogenes (c-Myc and Cdc25A). C-Myc scores were classified into 'low' (n=184) and 'high' (n=200) based on the median score. Also, Cdc25A scores were dichotomized into 'low' (n=202) and 'high' (n=182) groups based on the median score. Subsequently, c-Myc and CDC25A high/low subgroups were separately analyzed in breast

cancer subgroups ER/PR$^+$HER2$^-$, ER/PR$^+$HER2$^+$, ER/PR$^-$HER2$^+$ and TNBC. For all subgroups or total cohort, tumor expression of replication stress markers (pRPA and γ-H2AX) was assessed. Indicated *P*-values were calculated using Mann-Whitney U test. 'Ns' indicates not significant. * indicates $P<0.05$, ** indicates $P<0.01$, *** indicates $P<0.001$, **** indicates $P<0.0001$.

Supplemental Fig. 4: Associations between mRNA expression levels and survival in breast cancer.

(a) Disease-free survival of breast cancer patients of ER$^+$/HER2$^-$ (n=2153), ER$^+$/HER2$^+$ (n=341), ER$^-$/HER2$^+$ (n=291) and ER$^-$/HER2$^-$ (n=665) breast cancer subgroups. **(b)** Overall survival of breast cancer patients of ER$^+$/HER2$^-$ (n=807), ER$^+$/HER2$^+$ (n=165), ER$^-$/HER2$^+$ (n=192) and TNBC (n=263) breast cancer subgroups.

Supplemental Fig. 5: Uncropped Western blots.

Supplemental Table legends:

Supplemental Table 1: T-Test of MDA-MB231 survival in response to ATR or WEE1 inhibition.

Indicated P-values were calculated using two-tailed Student's t-test.

Supplemental Table 2: Clinicopathological characteristics of the study population. Overview of baseline clinical, pathological and treatment characteristics of patients from the study population cohort (n=384) and breast cancer molecular subgroups ER/PR^{+}HER2^{-} (n=161), ER/PR^{+}HER2^{+} (n=90), ER/PR^{-}HER2^{+} (n=27) and TNBC (n=106). The indicated *P*-values for tumor stage, tumor grade, radiation therapy, chemotherapy and endocrine therapy were obtained using Pearson Chi-Square tests, whereas the difference in age was assessed using a Kruskal-Wallis test.

Supplemental Table 3: Relation between breast cancer subgroups and clinicopathological characteristics in the study population.

The indicated *P*-values were obtained using a Pearson Chi-Square test. The *P*-values for age were assessed using a Mann-Whitney U test.

Supplemental Table 4: List of antibodies and protocols for immunohistochemical analysis. Information regarding antibodies, antigen retrieval methods and detection kits is presented.

Supplemental Table 5: Tumor expression of oncogenes and replication stress markers in the study population. Tumor expression of oncogenes CDC25A, Cyclin E (n), Cyclin E (c) c-Myc and the replication stress markers pRPA and γ-H2AX in the combined cohort (n=384) and breast cancer subgroups $ER/PR^{+}HER2^{-}$ (n=161), $ER/PR^{+}HER2^{+}$ (n=90), $ER/PR^{-}HER2^{+}$ (n=27) and TNBC (n=106).

Supplemental Table 6: Tumor expression of replication stress markers and oncogenes in relation to Androgen receptor status in TNBC.

(a) Tumor expression of replication stress markers pRPA and γ-H2AX in the combined TNBC cohort (n=106), and the AR^{-} (n=77) and AR^{+} (n=29) TNBC subgroups. **(b)** Tumor expression of oncogenes Cyclin E (n), Cyclin E (c), c-Myc and Cdc25A, in the combined TNBC cohort (n=106), and the AR^{-} (n=77) and AR^{+} (n=29) TNBC subgroups.

Supplemental Table 7: Spearman rank correlation of replication stress marker expression versus oncogene expression in the AR^{-}/AR^{+} TNBC subgroups.

(a) Association analysis between oncogene expression and pRPA expression in the combined TNBC cohort (n=106) and the indicated TNBC subgroups. **(b)** Association

analysis between oncogene expression and γ-H2AX expression in the combined TNBC cohort (n=106) and the indicated TNBC subgroups.

Supplemental Table 8: Associations between *CCNE1* mRNA expression and survival. Multivariate Cox regression analyses of *CCNE1* mRNA expression and survival corrected for age, tumor size, tumor grade, lymph node involvement, ER status, HER2 status, and treatment regimen on $ER/PR^{+}HER2^{-}$ (n=576), $ER/PR^{+}HER2^{+}$ (n=81), $ER/PR^{-}HER2^{+}$ (n=69) and TNBC (n=120) breast cancer patients.

Supplemental Figure 1

a

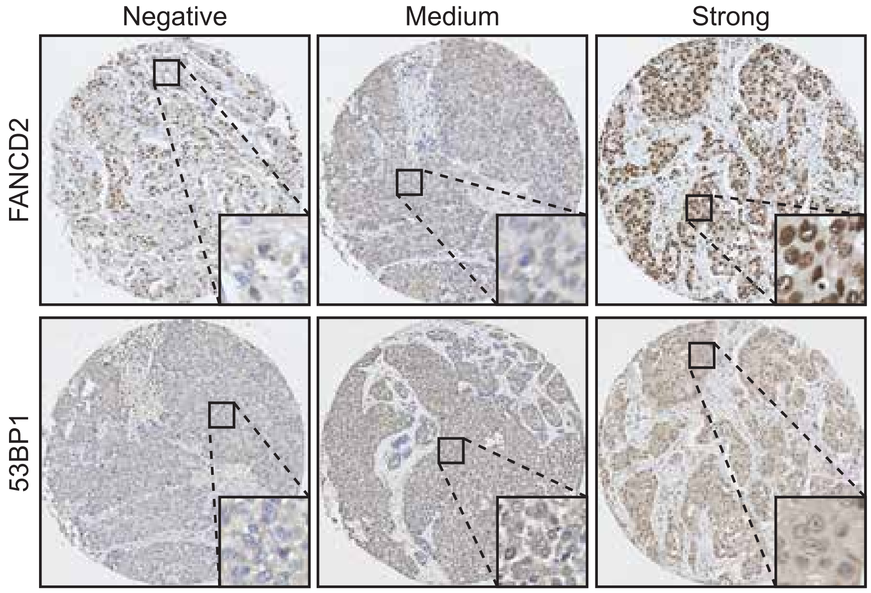

b

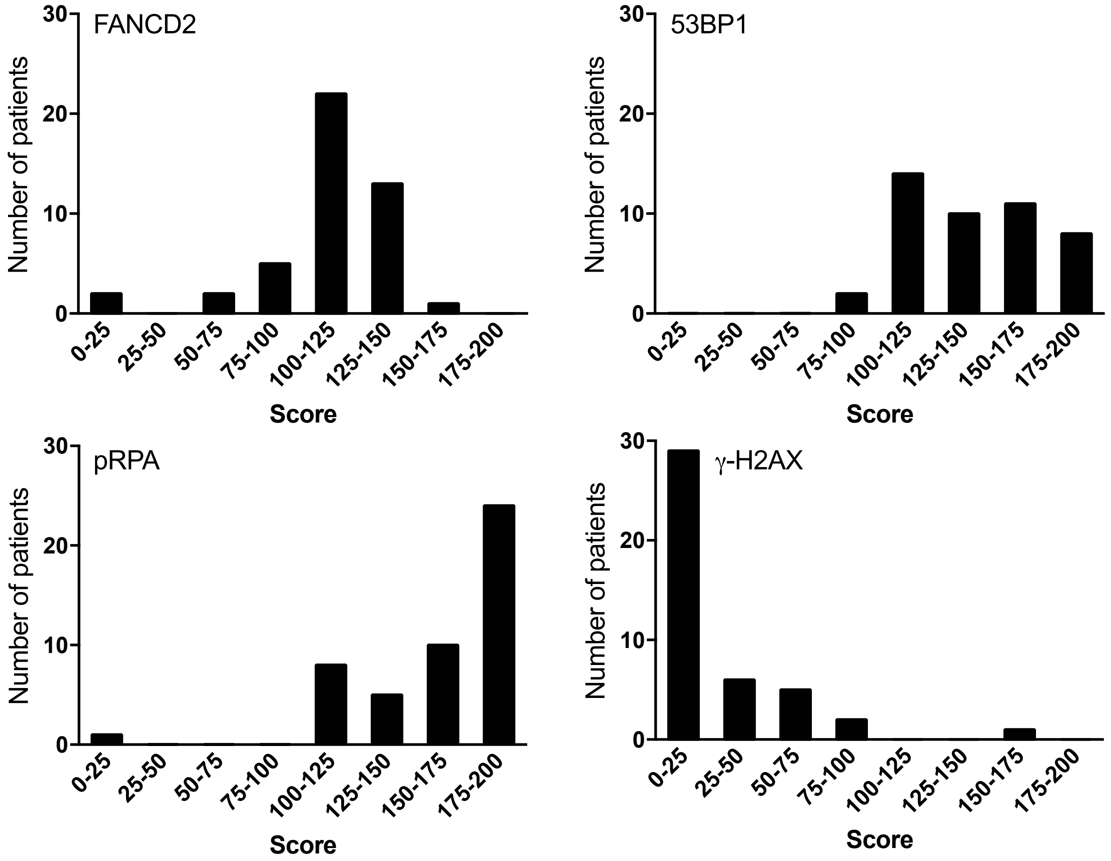

c

Spearman correlation analysis

variable	FANCD2	53BP1
pRPA (n=45)		
correlation	0.122	0.107
P-value	0.423	0.483

Spearman correlation analysis

variable	FANCD2	53BP1
γ-H2AX (n=41)		
correlation	0.118	0.344
P-value	0.462	0.028

Supplemental Figure 2

a

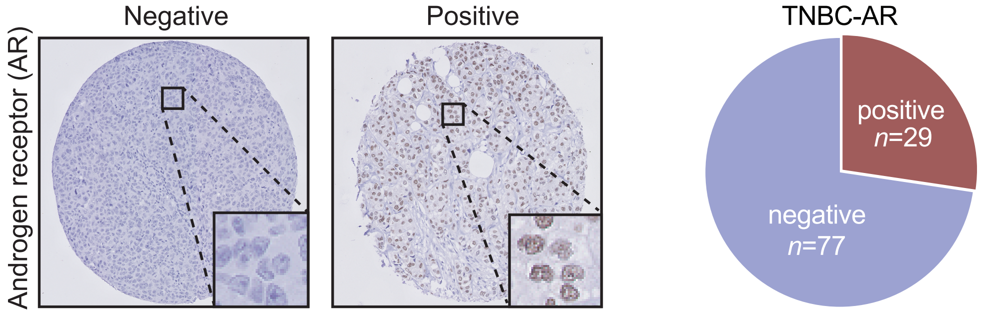

b

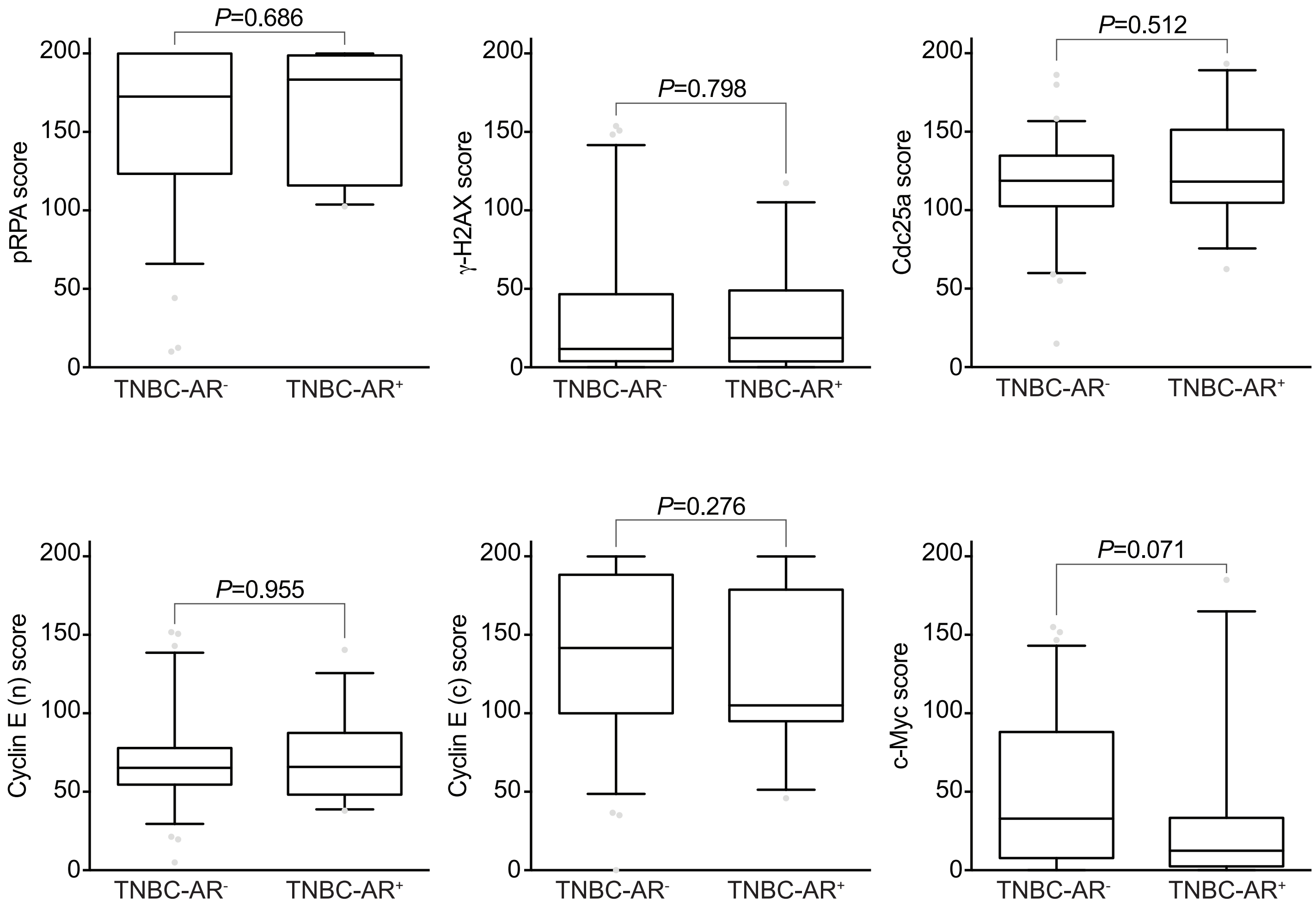

Supplemental Figure 3

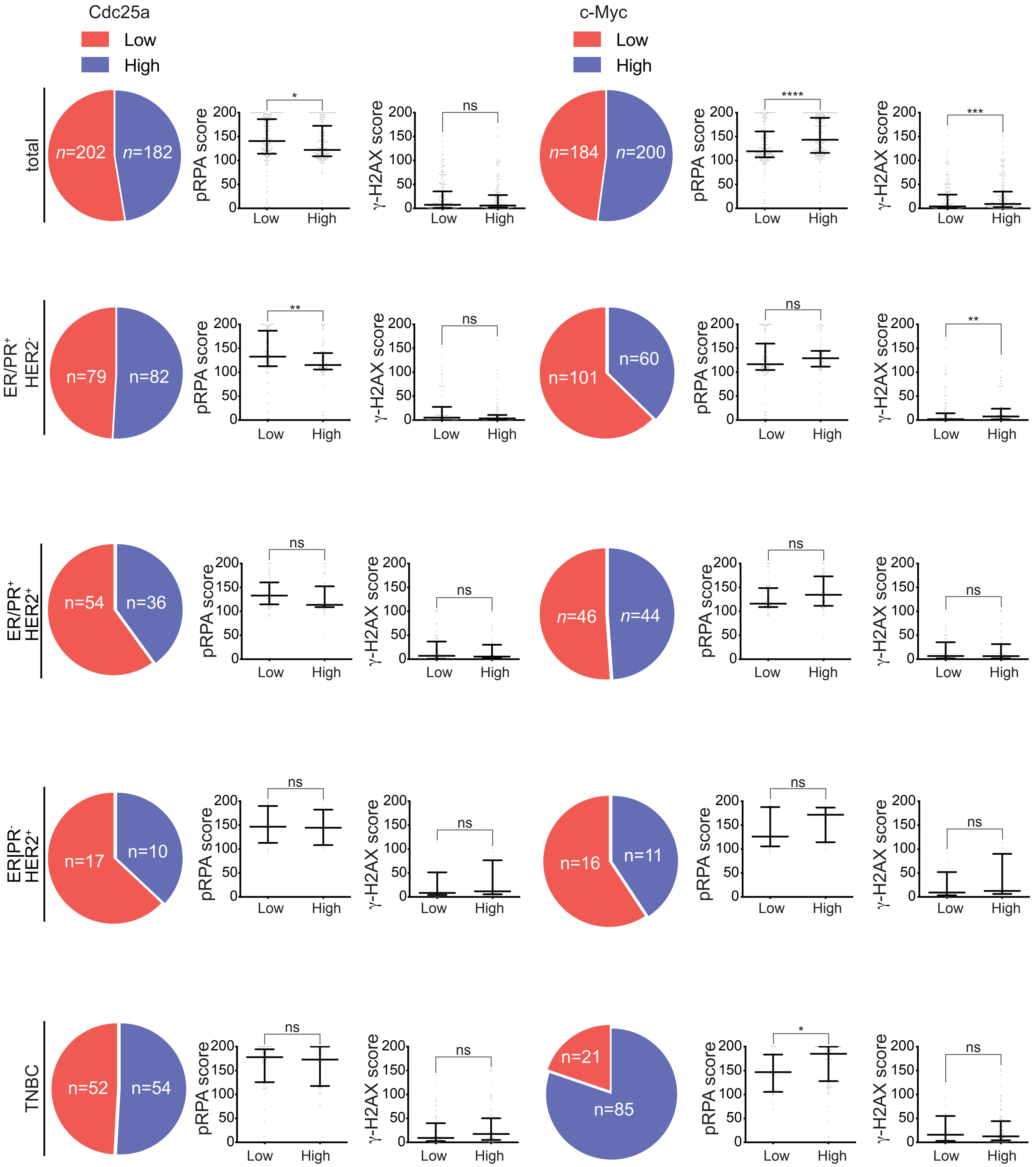

Supplemental Figure 4

a

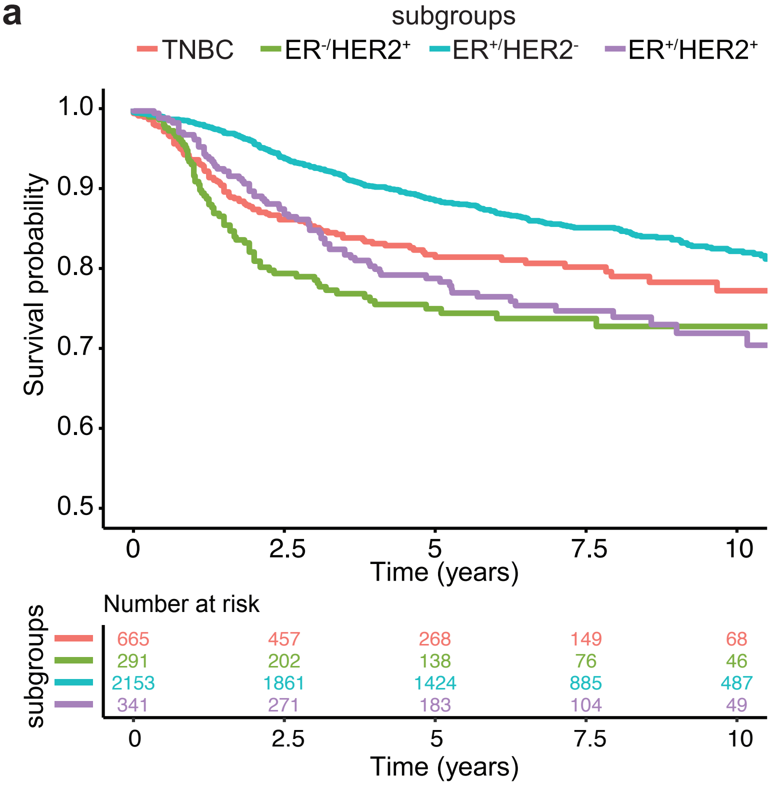

b

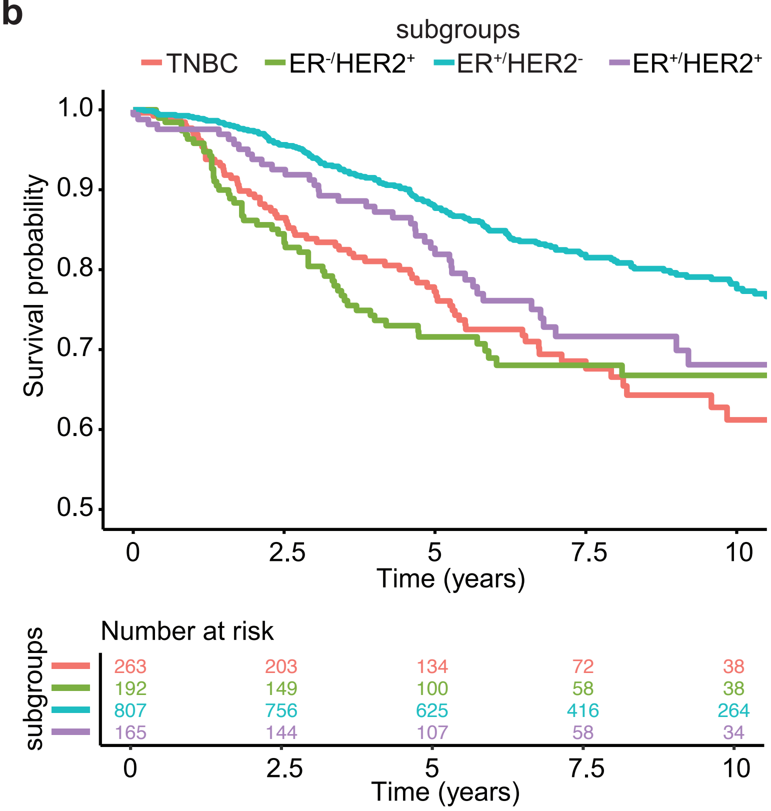

Supplemental Figure 5

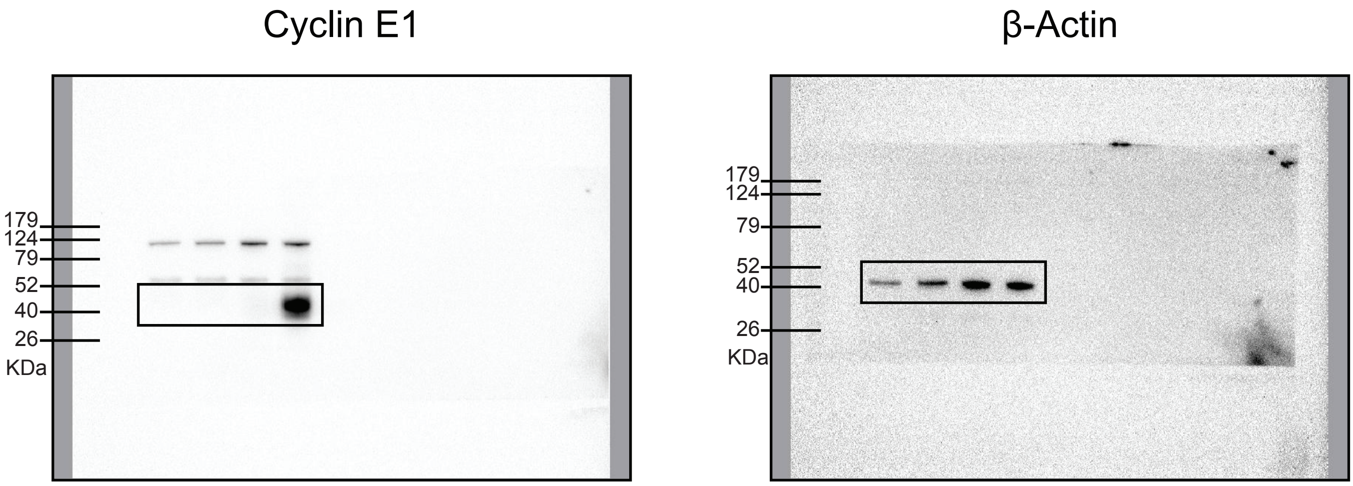

non-cropped Western blots of Figure 1a

Supplemental Table 1. Statistical analysis of cytotoxic effects in MDA-MB231 cells in response to ATR or WEE1 inhibition (Students T-test). Related to Figure 1D.

	**MDA-MB231 cells (*P*-value)**	
**VE-822 (µM)**	**Control^{-dox} vs Control^{+dox}**	**Cyclin E1^{-dox} vs Cyclin E1^{+dox}**
0	0.3081	0.1288
0.05	0.1017	0.0025
0.1	0.7008	<0.0001
0.2	0.0149	0.0009
0.4	0.0706	0.1211
0.8	0.4338	0.7293
1.6	0.9051	0.5115
3.2	0.5118	0.7212
**MK-1775 (µM)**	**Control^{-dox} vs Control^{+dox}**	**Cyclin E1^{-dox} vs Cyclin E1^{+dox}**
0	0.0898	0.6318
0.02	0.0709	0.9891
0.04	0.0883	0.1775
0.08	0.1281	0.0154
0.16	0.0118	<0.0001
0.32	0.2785	0.9166
0.64	0.2890	0.6318
1.28	0.1017	0.9600

Supplemental Table 2. Clinicopathological characteristics of the study population

	No. of patients (%)					
**Variable**	**Combined cohort n=384**	**ER/PR^{+} HER2^{-} n=161**	**ER/PR^{+} HER2^{+} n=90**	**ER/PR^{-} HER2^{+} n=27**	**TNBC n=106**	***P*-value**
Age (years)						0.036
Median	56.1	56.7	58.1	56.1	52.7	
Range	26.6-90.5	27.0-88.6	35.9-89.3	31.4-74.7	26.6-90.5	
T stage						0.040
T1	210 (54.7)	101 (62.7)	43 (47.8)	13 (48.1)	53 (50.0)	
T2	147 (38.3)	51 (31.7)	41 (45.6)	13 (48.1)	42 (39.6)	
T3	24 (6.3)	7 (4.3)	6 (6.7)	0 (0.0)	11 (10.4)	
NA	3 (0.8)	2 (1.2)	0 (0.0)	1 (3.7)	0 (0.0)	
Tumor grade						$1.72*10^{-13}$
I	70 (18.2)	44 (27.3)	17 (18.9)	4 (14.8)	5 (4.7)	
II	143 (37.2)	77 (47.8)	38 (42.2)	10 (37.0)	18 (17.0)	
III	169 (44.0)	40 (24.8)	35 (38.9)	13 (48.1)	81 (76.4)	
Unknown	2 (0.5)	0 (0.0)	0 (0.0)	0 (0.0)	2 (1.9)	
Radiation therapy						
No	174 (45.3)	58 (36.0)	41 (45.6)	7 (25.9)	68 (64.2)	$1.60*10^{-5}$
Yes	210 (54.7)	103 (64.0)	49 (54.4)	20 (74.1)	38 (35.8)	
Chemotherapy						0.079
No	167 (43.5)	79 (49.1)	42 (46.7)	10 (37.0)	36 (34.0)	
Yes	217 (56.5)	82 (50.9)	48 (53.3)	17 (63.0)	70 (66.0)	
Endocrine therapy						$3.58*10^{-10}$
No	268 (69.8)	93 (57.8)	55 (61.1)	19 (70.4)	101 (95.3)	
Yes	116 (30.2)	68 (42.2)	35 (38.9)	8 (29.6)	5 (4.7)	

Supplemental Table 3. Relation between breast cancer subtypes and clinicopathological characteristics in the study population

	Patient characteristics: *P*-value								
**BC subtype**	**ER/PR^+HER2^+**			**TNBC**			**ER/PR^-HER2^+**		
	**Age**	**T-Stage**	**T-Grade**	**Age**	**T-Stage**	**T-Grade**	**Age**	**T-Stage**	**T-Grade**
ER/PR^+HER2^-	0.358	0.072	0.052	0.029	0.052	$2.91*10^{-16}$	0.434	0.195	0.040
ER/PR^+HER2^+				0.007	0.542	$3.48*10^{-7}$	0.244	0.160	0.683
TNBC							0.411	0.068	0.016

	Treatment: *P*-value								
**BC subtype**	**ER/PR^+HER2^+**			**TNBC**			**ER/PR^-HER2^+**		
	**Chemo**	**Radio**	**Endo**	**Chemo**	**Radio**	**Endo**	**Chemo**	**Radio**	**Endo**
ER/PR^+HER2^-	0.715	0.138	0.605	0.015	$7.00*10^{-6}$	$1.70*10^{-11}$	0.247	0.307	0.217
ER/PR^+HER2^+				0.070	0.009	$3.31*10^{-9}$	0.377	0.069	0.381
TNBC							0.764	$3.49*10^{-4}$	$1.00*10^{-4}$

Supplemental Table 4. Antibodies and antigen retrieval methods

Antibody	Clone	Supplier	Dilution	Antigen retrieval	Secondary antibody	Tertiary antibody
Cdc25A	144	Santa Cruz (sc-97)	1:400	Citrate (pH 6.0)	Envision kit anti-Rabbit (K4009)	-
Cyclin E	C19	Santa Cruz (sc-198)	1:1000	Citrate (pH 6.0)	GARbio (1:300)	STRpo (1:300)
c-Myc	Y69	Roche (790-4628)	RTU	CC1, Tris-based (pH 8.0-9.0)	Ultra View Universal DAB Detection Kit (760-500)	-
p-RPA	S33	Bethyl (A300-246A)	1:6400	CC2, Citrate-based (pH 6.0)	Ultra View Universal DAB Detection Kit (760-500)	-
γ-H2AX	JBW301	Millipore (05-636)	1:300	Citrate (pH 6.0)	RAMpo (1:100)	GARpo (1:100)
AR	SP107	Roche (760-4605)	RTU	CC1, Citrate-based (pH 6.0)	Ultra View Universal DAB Detection Kit (760-500)	
53-BP1	-	Bethyl (00001)	1:300	Citrate (pH 6.0)	GARpo (1:100)	RAGpo (1:100)
FANCD2	-	Bethyl (00624)	1:400	Tris/HCl (pH 9.0)	GARpo (1:100)	RAGpo (1:100)

RTU= Ready to use
RAMpo = Rabbit-anti-Mouse horseradish peroxidase
GARbio = Goat-anti-Rabbit-biotin
GARpo =Goat-anti-Rabbit-horseradish peroxidase
STRpo = Streptavidin-horseradish peroxidase

RAGpo=Rabbit-anti-Goat peroxidase

CC1= Cell Conditioning 1, Ventana

CC2= Cell Conditioning 2, Ventana

AR=Androgen Receptor

Supplemental Table 5. Tumor expression of Cdc25A, Cyclin E, c-Myc, pRPA and γ-H2AX in the study population

	TMA score				
**BC subgroup**	**Mean**	**SD**	**P5**	**P50**	**P95**
Cdc25A					
Combined cohort (n=384)	119.5	25.7	85	116.7	170
ER/PR^+HER2^- (n=161)	122.4	25.1	87.6	117.5	174.9
ER/PR^+HER2^+ (n=90)	116.5	24.5	86.4	110	170.4
ER/PR^-HER2^+ (n=27)	115	18	88.5	110	150
TNBC (n=106)	118.8	28.9	61.4	117.9	169.6
Cyclin E (n)					
Combined cohort (n=384)	54.1	21.4	32.3	48.3	97.5
ER/PR^+HER2^- (n=161)	49.1	16.1	32.6	43.9	84.8
ER/PR^+HER2^+ (n=90)	46.5	12.7	30.4	44.1	72.9
ER/PR^-HER2^+ (n=27)	48.5	14.8	30.9	47.2	80.5
TNBC (n=106)	69.4	27.5	34.2	65.3	134.3
Cyclin E (c)					
Combined cohort (n=384)	126.4	46.7	57.5	108.3	200
ER/PR^+HER2^- (n=161)	117.3	48.9	9.3	100	200
ER/PR^+HER2^+ (n=90)	128	37.7	89.6	110	200
ER/PR^-HER2^+ (n=27)	140.5	44.1	92.5	112.5	200
TNBC (n=106)	135.3	48.6	52.3	131.7	200
c-Myc					
Combined cohort (n=384)	24.1	40.4	0	3.3	131.3
ER/PR^+HER2^- (n=161)	15.1	32.6	0	0.8	95.7
ER/PR^+HER2^+ (n=90)	16.6	30.2	0	2.1	90
ER/PR^-HER2^+ (n=27)	14.7	33.9	0	2.5	127.3
TNBC (n=106)	46.5	50.8	0	25	144.4
pRPA					
Combined cohort (n=384)	139.1	44.7	69.4	132.5	200
ER/PR^+HER2^- (n=161)	128.2	45	19.9	122.5	200
ER/PR^+HER2^+ (n=90)	132	38	90.7	123.3	200
ER/PR^-HER2^+ (n=27)	147.8	38.5	94.5	146.7	200
TNBC (n=106)	159.4	44.3	76.2	175	200
γ-H2AX					
Combined cohort (n=384)	23.1	33.7	0	6.3	98.3
ER/PR^+HER2^- (n=161)	18.5	32.7	0	3.8	97.4
ER/PR^+HER2^+ (n=90)	20.6	27.4	0	6.5	85.5
ER/PR^-HER2^+ (n=27)	30.4	35.6	0	10.8	109.2
TNBC (n=106)	30.6	38.3	0	13.6	119.1

Supplemental Table 6a. Tumor expression of pRPA and γ-H2AX of TNBC in relation to AR staining

	TMA score				
**BC subtype**	**Mean**	**SD**	**P5**	**P50**	**P95**
pRPA					
Combined cohort (n=106)	159.4	44.3	76.2	175.0	200.0
TNBC-AR$^-$ (n=77)	157.5	46.6	65.9	172.5	200.0
TNBC-AR$^+$ (n=29)	164.7	37.6	103.8	183.3	200.0
γ-H2AX					
Combined cohort (n=106)	30.6	38.3	0.0	13.6	119.1
TNBC-AR$^-$ (n=77)	31.0	40.7	0.0	11.7	141.6
TNBC-AR$^+$ (n=29)	29.7	31.8	0.0	17.5	103.8

Supplemental Table 6b. Tumor expression of Cdc25A, Cyclin E and c-Myc of TNBC in relation to AR staining

	TMA score				
**BC subtype**	**Mean**	**SD**	**P5**	**P50**	**P95**
Cdc25A					
Combined cohort (n=106)	118.8	28.9	61.4	117.9	169.6
TNBC-AR$^-$ (n=77)	116.7	28.0	59.9	118.8	156.8
TNBC-AR$^+$ (n=29)	124.3	31.1	73.8	116.7	188.3
Cyclin E (n)					
Combined cohort (n=106)	69.4	27.5	34.2	65.3	134.3
TNBC-AR$^-$ (n=77)	69.3	28.2	29.5	65.3	138.6
TNBC-AR$^+$ (n=29)	69.8	25.8	38.8	65.8	125.6
Cyclin E (c)					
Combined cohort (n=106)	135.3	48.6	52.3	131.7	200.0
TNBC-AR$^-$ (n=77)	138.3	48.8	48.7	141.7	200.0
TNBC-AR$^+$ (n=29)	127.5	48.1	51.3	105.0	200.0
c-Myc					
Combined cohort (n=106)	46.5	50.8	0.0	25.0	144.4
TNBC-AR$^-$ (n=77)	51.0	49.9	0.0	32.5	142.9
TNBC-AR$^+$ (n=29)	34.6	51.9	0.0	12.5	165.0

Supplemental Table 7a. Spearman correlation test of Cdc25A, Cyclin E and c-Myc versus pRPA of TNBC

	BC Subtype		
	**TNBC (all)** n=106	**TNBC-AR$^-$** n=77	**TNBC-AR$^+$** n=29
**Variable**	**pRPA**		
Cdc25A			
Correlation	0.035	0.131	-0.214
P-value	0.719	0.256	0.265
Cyclin E (n)			
Correlation	0.432	0.463	0.327
P-value	$4.00*10^{-6}$	$2.20*10^{-5}$	0.083
Cyclin E (c)			
Correlation	0.262	0.320	0.092
P-value	0.007	0.005	0.637
c-Myc			
Correlation	0.359	0.360	0.335
P-value	$1.58*10^{-4}$	0.001	0.076

Supplemental Table 7b. Spearman correlation test of Cdc25A, Cyclin E and c-Myc versus γ-H2AX of TNBC

	BC Subtype		
	**TNBC (all)** n=106	**TNBC-AR$^-$** n=77	**TNBC-AR$^+$** n=29
**Variable**	**γ-H2AX**		
Cdc25A			
Correlation	0.170	0.275	-0.100
P-value	0.081	0.016	0.607
Cyclin E (n)			
Correlation	0.705	0.755	0.574
P-value	$3.53*10^{-17}$	0.016	0.001
Cyclin E (c)			
Correlation	0.078	0.118	0.048
P-value	0.424	0.306	0.806
c-Myc			
Correlation	-0.032	-0.028	0.070
P-value	0.743	0.808	0.718

Supplemental Table 8. Associations between *CCNE1* mRNA expression and survival

	Multivariate			
**Disease-free survival**	**Events**	**HR**	**95% CI**	***P*-value**
All BC (n=846)	147	1.070	0.840-1.370	0.584
ER/PR$^+$HER2$^-$ (n=576)	97	1.290	0.970-1.720	0.075
ER/PR$^+$HER2$^+$ (n=81)	18	0.930	0.400-2.160	0.870
ER/PR$^-$HER2$^+$ (n=69)	13	0.440	0.170-1.170	0.099
TNBC (n=120)	19	0.980	0.530-1.820	0.949
**Overall survival**	**Events**	**HR**	**95% CI**	***P*-value**
All BC (n=632)	153	1.090	0.850-1.420	0.492
ER/PR$^+$HER2$^-$ (n=417)	87	1.660	1.170-2.350	0.004
ER/PR$^+$HER2$^+$ (n=55)	20	0.750	0.180-3.100	0.693
ER/PR$^-$HER2$^+$ (n=64)	19	0.740	0.360-1.520	0.413
TNBC (n=96)	27	0.870	0.510-1.460	0.589
