# Supplementary material for: Cyclin E expression is associated with high levels of replication stress in triple-negative breast cancer
Source: NPJ Breast Cancer. 2020 Sep 7;6:40. doi: 10.1038/s41523-020-00181-w (PMC7477160; doi:10.1038/s41523-020-00181-w)
Supplement: Supplementary file 2 — Reporting Summary [file 41523_2020_181_MOESM2_ESM.pdf]

nature research

Corresponding author(s): Marcel A.T.M. van Vugt

Last updated by author(s): Feb 24, 2020

Reporting Summary

Nature Research wishes to improve the reproducibility of the work that we publish. This form provides structure for consistency and transparency in reporting. For further information on Nature Research policies, see Authors & Referees and the Editorial Policy Checklist.

Statistics

For all statistical analyses, confirm that the following items are present in the figure legend, table legend, main text, or Methods section.

n/a	Confirmed	
☐	☒	The exact sample size (*n*) for each experimental group/condition, given as a discrete number and unit of measurement
☐	☒	A statement on whether measurements were taken from distinct samples or whether the same sample was measured repeatedly
☐	☒	The statistical test(s) used AND whether they are one- or two-sided<br>*Only common tests should be described solely by name; describe more complex techniques in the Methods section.*
☐	☒	A description of all covariates tested
☐	☒	A description of any assumptions or corrections, such as tests of normality and adjustment for multiple comparisons
☐	☒	A full description of the statistical parameters including central tendency (e.g. means) or other basic estimates (e.g. regression coefficient) AND variation (e.g. standard deviation) or associated estimates of uncertainty (e.g. confidence intervals)
☒	☐	For null hypothesis testing, the test statistic (e.g. *F*, *t*, *r*) with confidence intervals, effect sizes, degrees of freedom and *P* value noted<br>*Give P values as exact values whenever suitable.*
☒	☐	For Bayesian analysis, information on the choice of priors and Markov chain Monte Carlo settings
☒	☐	For hierarchical and complex designs, identification of the appropriate level for tests and full reporting of outcomes
☒	☐	Estimates of effect sizes (e.g. Cohen's *d*, Pearson's *r*), indicating how they were calculated

Our web collection on statistics for biologists contains articles on many of the points above.

Software and code

Policy information about availability of computer code

Data collection	na
Data analysis	na

For manuscripts utilizing custom algorithms or software that are central to the research but not yet described in published literature, software must be made available to editors/reviewers. We strongly encourage code deposition in a community repository (e.g. GitHub). See the Nature Research guidelines for submitting code & software for further information.

Data

Policy information about availability of data

All manuscripts must include a data availability statement. This statement should provide the following information, where applicable:

- Accession codes, unique identifiers, or web links for publicly available datasets
- A list of figures that have associated raw data
- A description of any restrictions on data availability

The data that support the findings of this study are available from the corresponding author upon reasonable request. Uncropped Western blots from figure 1 are presented in Supplemental Figure 5.

Field-specific reporting

Please select the one below that is the best fit for your research. If you are not sure, read the appropriate sections before making your selection.

☒ Life sciences ☐ Behavioural & social sciences ☐ Ecological, evolutionary & environmental sciences

Life sciences study design

All studies must disclose on these points even when the disclosure is negative.

Sample size	no power calculation was performed. We have used samples based on availability. In testing associations of protein expression versus progression-free or overall survival, known relevant co-variates were included. These co-variates (Age, stage, etc) confirmed known associates and provide a rationale for using this sample size.
Data exclusions	no data was excluded, except when tumor stainings could not be quantified on three separates cores in our tissue microarrays, as stated in our methods and materials section.
Replication	replication of tumor stainings are taken into account in the requirement that three cores of a tumor need to be quantifiable. Scoring of immunohostochemistry was done in a blinded fashion, by two independent persons, and inconclusive scoring was discussed until consensus. MTT data in figure 1 was based on 6 replicates.
Randomization	no randomization was performed.
Blinding	Scoring of immunohostochemistry was done in a blinded fashion, by two independent persons.

Reporting for specific materials, systems and methods

We require information from authors about some types of materials, experimental systems and methods used in many studies. Here, indicate whether each material, system or method listed is relevant to your study. If you are not sure if a list item applies to your research, read the appropriate section before selecting a response.

Materials & experimental systems

n/a	Involved in the study	
☐	☒	Antibodies
☐	☒	Eukaryotic cell lines
☐	☐	Palaeontology
☐	☐	Animals and other organisms
☐	☐	Human research participants
☐	☒	Clinical data

Methods

n/a	Involved in the study	
☒	☐	ChIP-seq
☒	☐	Flow cytometry
☒	☐	MRI-based neuroimaging

Antibodies

Antibodies used

anti-Cdc25A (1:400; rabbit, #sc-97, clone 144; Santa Cruz Biotechnology, CA, USA).
anti-Cyclin E (1:1000; rabbit, #sc-198, clone C19; Santa Cruz Biotechnology, CA, USA)
anti-c-Myc (RTU; rabbit, #790-4628, clone Y69; Roche, Basel, Switzerland)
anti-phospho-RPA32 (Ser33) (1:6400; rabbit, #A300-246A, clone S33; Bethyl, Texas, USA)
anti-γ-H2AX (1:300; mouse, #05-636, clone JBW301; Millipore, Amsterdam, The Netherlands)
anti-androgen receptor (AR) (RTU; rabbit, #760-4605, clone SP107; Roche, Basel, Switzerland)
anti-53BP1(1:300; rabbit, #IHC-00001; Bethyl, Texas, USA)
anti-FANCD2 (1:400; rabbit, #IHC-00624; Bethyl, Texas, USA)

Validation

All staining protocols were validated with control IgG stainings, which did not show background signal.

Anti-Cdc25A (144) is an affinity purified rabbit polyclonal antibody raised against
a peptide mapping near the C-terminus of Cdc25A of human origin. This antibody has been tested for IHC of paraffin-embedded material by provider.

Anti-cyclin E (C-19) is available as either rabbit (sc-198) or goat (sc-198-G) polyclonal affinity purified antibody raised against a peptide mapping at the C-terminus of cyclin E of human origin. The antibody is tested and recommended for IHC of paraffin-embedded material by provider.

Anti-c-MYC (Y69) Rabbit Monoclonal Primary Antibody (anti-c-MYC (Y69)) is directed against the transcription factor c-MYC, an important factor in cell cycle regulation. The anti-c-MYC (Y69) antibody exhibits a nuclear staining pattern and may be used to aid in the characterization of lymphoma. This antibody is intended for qualitative staining of sections of formalin-fixed, paraffin-embedded tissue.

Anti-phospho-Histone H2A.X (Ser139), clone JBW301 is a well published Mouse Monoclonal Antibody validated in ChIP, ICC, IF, WB. This purified mAb is highly specific for phospho-Histone H2A.X (Ser139) also known as H2AXS139p.

The anti-53BP1 antibody was affinity purified using an epitope specific to 53BP1 immobilized on solid support.
The epitope recognized by IHC-00001 maps to a region between residue 350 and 400 of human

tumor protein p53 binding protein 1 using the numbering given in entry NP_005648.1 (GeneID 7158). This antibody is intended for staining of sections of formalin-fixed, paraffin-embedded tissue according to provider.

anti-FANCD2 antibody was affinity purified using an epitope specific to FANCD2 immobilized on solid support. The epitope recognized by A302-174A maps to a region between residue 1401 and 1451 of human Fanconi anemia, complementation group D2 using the numbering given in entry NP_001018125.1 (GeneID 2177). This antibody is intended for staining of sections of formalin-fixed, paraffin-embedded tissue according to provider.

phospho-RPA32: Immunogen was a phosphorylated synthetic peptide, which represented a portion of human replication protein A2, 32 kDa surrounding phosphorylated serine that corresponded to position 33 using the numbering given in entry NP_002937.1 (GeneID 6118). Antibody was affinity purified using the peptide immobilized on solid support. The antibody was tested for use in IHC by treating HeLa cells with/without ATR inhibitor and subsequent fixation of cell pellets in formalin and embedding of cell pellets in paraffin.

Eukaryotic cell lines

Policy information about cell lines

Cell line source(s)	MDA-MB-231 cells were derived from ATCC
Authentication	MDA-MB-231 cell line was authenticated using STR profiling
Mycoplasma contamination	MDA-MB-231 cell line was tested negative for mycoplasm
Commonly misidentified lines (See ICLAC register)	not applicable

Palaeontology

Specimen provenance	na
Specimen deposition	na
Dating methods	na

☐ Tick this box to confirm that the raw and calibrated dates are available in the paper or in Supplementary Information.

Animals and other organisms

Policy information about studies involving animals; ARRIVE guidelines recommended for reporting animal research

Laboratory animals	na
Wild animals	na
Field-collected samples	na
Ethics oversight	na

Note that full information on the approval of the study protocol must also be provided in the manuscript.

Human research participants

Policy information about studies involving human research participants

Population characteristics	na
Recruitment	na
Ethics oversight	na

Note that full information on the approval of the study protocol must also be provided in the manuscript.

Clinical data

Policy information about clinical studies

All manuscripts should comply with the ICMJE guidelines for publication of clinical research and a completed CONSORT checklist must be included with all submissions.

Clinical trial registration	na. our analysis of clinical data was performed on archieval material of pathology material, and not part of a registered clinical study.
Study protocol	na
Data collection	anonimized clinical data were retrieved from our patient registry
Outcomes	overall survival, progression-free survival and recurrence -free survival were derived from clinical records.
